# Antcin-B, a phytosterol-like compound from *Taiwanofungus camphoratus* inhibits SARS-CoV-2 3-chymotrypsin-like protease (3CL^Pro^) activity in silico and in vitro

**DOI:** 10.1038/s41598-023-44476-x

**Published:** 2023-10-10

**Authors:** Gyaltsen Dakpa, K. J. Senthil Kumar, Jochem Nelen, Horacio Pérez-Sánchez, Sheng-Yang Wang

**Affiliations:** 1 Molecular and Biological Agricultural Sciences Program, Taiwan International Graduate Program, Academia Sinica, Taipei, 108 Taiwan; 2 Graduate Institute of Biotechnology, National Chung Hsing University, Taichung, 402 Taiwan; 3 Bachelor Program of Biotechnology, National Chung Hsing University, Taichung, 402 Taiwan; 4 Structural Bioinformatics and High-Performance Computing Research Group (BIO-HPC), HiTech Innovation Hub, Universidad Católica de Murcia (UCAM), 30107 Murcia, Spain; 5 Department of Forestry, National Chung Hsing University, Taichung, 402 Taiwan; 6 Special Crop and Metabolome Discipline Cluster, Academy of Circle Economy, National Chung Hsing University, Taichung, 402 Taiwan; 7 Agricultural Biotechnology Research Center, Academia Sinica, Taipei, 108 Taiwan

**Keywords:** HIV infections, Enzymes, Chemical biology, Drug discovery

## Abstract

Despite the remarkable development of highly effective vaccines, including mRNA-based vaccines, within a limited timeframe, coronavirus disease 2019 (COVID-19) caused by severe acute respiratory syndrome coronavirus 2 (SARS-CoV-2) is not been entirely eradicated. Thus, it is crucial to identify new effective anti-3CL^Pro^ compounds, pivotal for the replication of SARS-CoV-2. Here, we identified an antcin-B phytosterol-like compound from *Taiwanofungus camphoratus* that targets 3CL^Pro^ activity. MTT assay and ADMET prediction are employed for assessing potential cytotoxicity. Computational molecular modeling was used to screen various antcins and non-antcins for binding affinity and interaction type with 3CL^Pro^. Further, these compounds were subjected to study their inhibitory effects on 3CL^Pro^ activity in vitro. Our results indicate that antcin-B has the best binding affinity by contacting residues like Leu141, Asn142, Glu166, and His163 via hydrogen bond and salt bridge and significantly inhibits 3CLPro activity, surpassing the positive control compound (GC376). The 100 ns molecular dynamics simulation studies showed that antcin-B formed consistent, long-lasting water bridges with Glu166 for their inhibitory activity. In summary, antcin-B could be useful to develop therapeutically viable drugs to inhibit SARS-CoV-2 replication alone or in combination with medications specific to other SARS-CoV-2 viral targets.

## Introduction

The severe acute respiratory syndrome coronavirus 2 (SARS-CoV-2) has caused one of the most devastating pandemics in modern history, posing a significant threat to global health and economic stability and endangering countless lives worldwide^1,2^. Despite the remarkable development of highly effective vaccines, including mRNA-based vaccines, within a limited time frame^3^, the pandemic has not been completely eradicated. Numerous strategies have been employed to combat SARS-CoV-2 infection, such as developing drugs targeting the spike protein and the creation of vaccines that stimulate the production of virus-specific antibodies by the immune system. Among these approaches, blocking or inhibiting the SARS-CoV-2 3C-like protease (3CL^Pro^) has emerged as a highly validated method for designing antivirals against coronaviruses. Approximately 70% of the SARS-CoV-2 genome encodes two polyproteins, pp1a, and pp1ab, which undergo proteolytic cleavage, generating 16 non-structural proteins (nsps)^1^. This cleavage process is facilitated by two proteases, nsp3, and nsp5, commonly referred to as 3-chymotrypsin-like protease (3CL^Pro^) or the main protease (MPro). These proteases play a crucial role in viral replication, modulation of polymerase fidelity, and regulation of nucleotide incorporation within host organisms^4^. Therefore, inhibition of 3CL^Pro^ prevents the release of nsp4 to nsp16, which halts viral replication within the host organism.

The SARS-CoV-2-3CL^Pro^ is a cysteine protease that contains a catalytic dyad consisting of His41 and Cys145, which are highly conserved residues^5^. The native structure of 3CL^Pro^ is a homodimer composed of domains (domains I, II, and III). Among them, domain III is necessary for proteolysis, as the protease is active only in the dimeric conformation^6^. 3CL^Pro^ is a highly conserved sequence, and essential functional properties make it a potential target for developing drugs to treat SARS-CoV, MERS, and SARS-CoV-2^7,8^. When the substrate binds to the enzyme, His41 deprotonates the thiol side chain of Cys145, facilitating its nucleophilic attack on the peptide substrate^5^. The deprotonation of Cys145 enables a nucleophilic attack on the carbonyl carbon of glutamine in the polyprotein backbone, resulting in the formation of a tetrahedral thiohemiacetal intermediate and oxyanion. His41 stabilizes the oxyanion through nearby hydrogen bond donating amide groups of Gys143 and Ser144. Ultimately, this process leads to the cleavage of the peptide bond and the release of the C-terminal part of the polyprotein substrate^9^. Thus, several studies have reported that natural and synthetic compounds targeting SARS-CoV-2-3CL^Pro^ are covalent compounds derived from peptidic scaffolds with an electrophile that reacts with the catalytic cysteine Cys145 and His41^10–15^. However, inhibitors derived from *Taiwanofungus camphoratus* target catalytic sites using other bonds such as hydrogen, hydrophobic interaction, and salt bridge to block the protease activities have yet to be reported.

**Figure 1:**
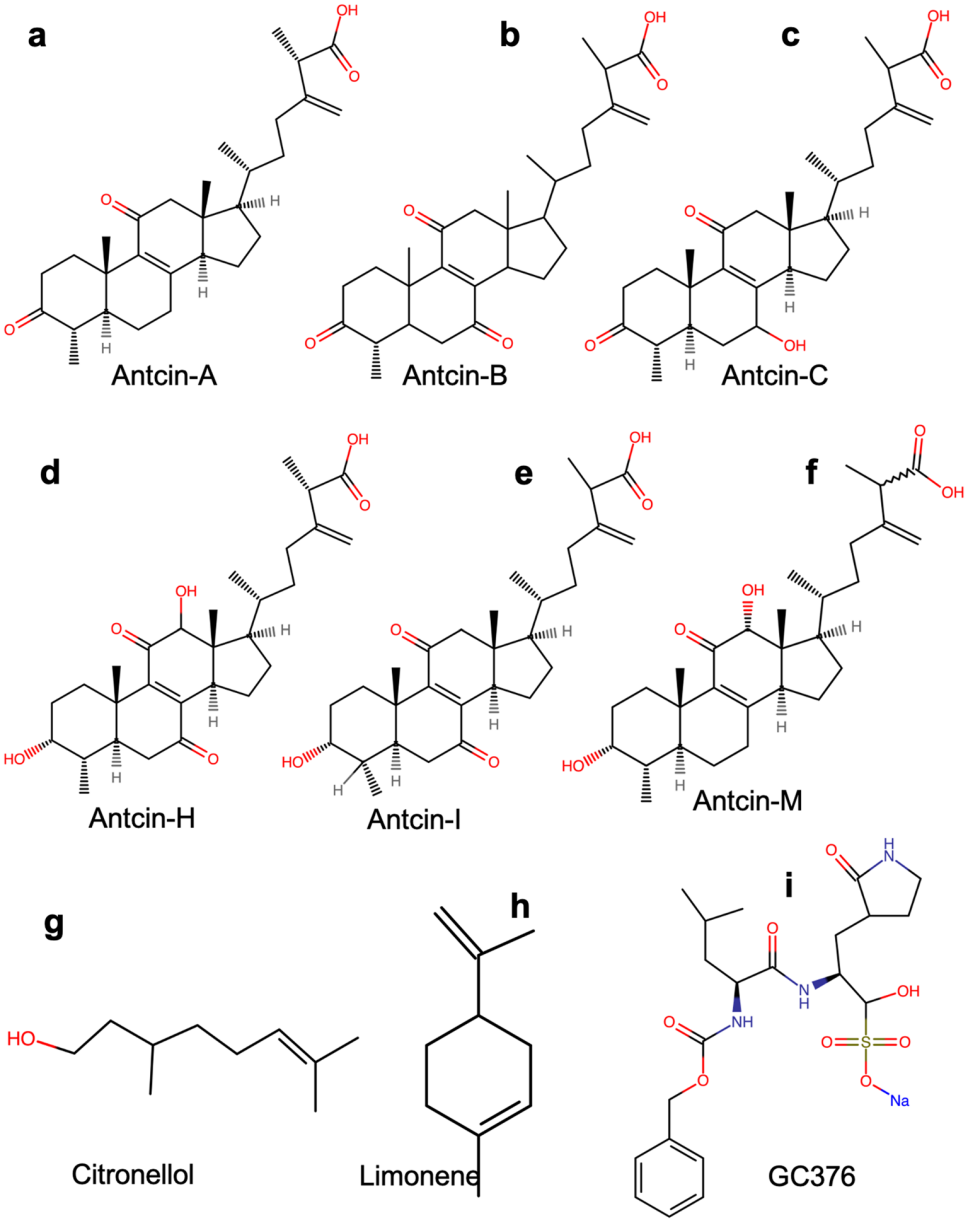
Chemical structures of natural compounds to test inhibition of SARS-CoV-2-3CL^Pro^. The chemical structure of (**a**) antcin-A, (**b**) antcin-B, (**c**) antcin-C, (**d**) antcin-H, (**e**) antcin-I, (**f**) antcin-M, (**g**) citronellol, (**h**) limonene, and (**i**) GC367. Relatively, all antcins structurally resemble the backbone except for a few functional groups. Non-antcins have different chemical structures compared to antcins. However, as shown in the figure, GC376 has benzene as the backbone and other functional groups.

*Taiwanofungus camphoratus* (syn. *Antrodia cinnamomea, Antrodia camphorata*) and *Antrodia salmonea* are edible medicinal fungi belonging to Agaricomycetes and order Polyporales. These fungi produce steroid-like compounds called antcins^16,17^. Traditional medicinal uses of *T. camphoratus* include the treatment of liver diseases, drug intoxication, diarrhea, abdominal pain, hypertension, and tumorigenic diseases^18^. There have been 18 antcins isolated from *T. camphoratus*. Antcin-A, antcin-B, antcin-C, antcin-H, antcin-I, and antcin-M (Fig. 1) are the most abundant and significantly valued for therapeutic drug applications^16^. Our recent studies have shown that antcins, citronellol, and limonene significantly reduce the expression of ACE2 receptors, facilitating the entry of SARS-CoV-2 into cells^19^. Furthermore, antcin-A regulates metabolomic pathways altered by the SARS-CoV-2 spike protein in the THP-1 cells, as observed through ^1^H-NMR metabolomic analysis^20^. These findings suggest that antcins can potentially develop therapeutic drugs against COVID-19. However, further scientific evidence is still needed to determine the role of antcins (A, B, C, H, I, and M) in inhibiting the activity of SARS-CoV-2-3CL^Pro^, which is crucial for viral replication in host cells and represents an effective target for COVID-19 treatment. Therefore, our study aims to investigate the role of antcins isolated from *T. camphoratus* on SARS-CoV-2-3CL^Pro^ enzyme activity. This effort further supports the discovery of the potential inhibitory activity of antcins on SARS-CoV-2-3CL^Pro^ and their viability as novel therapeutic strategies for COVID-19.

In silico virtual screening, docking, and molecular dynamics (MD) simulation investigations represent expedient methodologies renowned for their rapidity and quantity, which reduce the time consumed for experiments, the cost associated with drug discovery, increase predictive accuracy in binding poses of ligands and a target protein, and provide detailed information on the stability of the protein–ligand complex^21,22^. These techniques have contributed tremendously to the discovery of compounds with the potential for anti-SARS-CoV-2, including the anti-RNA methyltransferase that screened more than 5000 natural compounds at once^23^, the discovery of binding sites and structural features of SARS-CoV-2, which led to an understanding of the increased differential infectivity of omicron^24^ and even predicted the efficacy of antibodies with double mutations in the receptor binding domain (RBD) of the SARS-CoV-2 spike protein^25^. Subsequently, cell-based or enzyme assays can validate the predictions of computational techniques. Thus, virtual and in vitro screening is ideal for developing novel drugs that inhibit specific protein activity^26^. Therefore, we employed in silico and in vitro methods to discover the potential role of antcins isolated from *T. camphoratus* in inhibiting SARS-CoV-2-3CL^pro^ activity against COVID-19. CB-Dock2^27^ [https://cadd.labshare.cn/cb-dock2/php/index.php] and UCSF Chimera^28^ were used for docking, which predicted a success rate of more than 85% compared to FitDock, MTiAutoDock, SwissDock, and COACH-Dn^27^. Protein–Ligand Interaction Profiler (PLIP)^29^ [https://plip-tool.biotec.tu-dresden.de/plip-web/plip/index] and Protein*Plus*^30^ [https://proteins.plus] were used for chemical interactions to reveal polar and non-polar interactions. The PyMOL Molecular Graphics System was employed to visualize detailed chemical interactions in the ligand–protein complex. Simultaneously, Maestro-Desmond was used as software to perform MD simulations to study the detailed stability of the protein–ligand interactions.

## Results

### Antcins have less cytotoxicity than GC376

**Figure 2:**
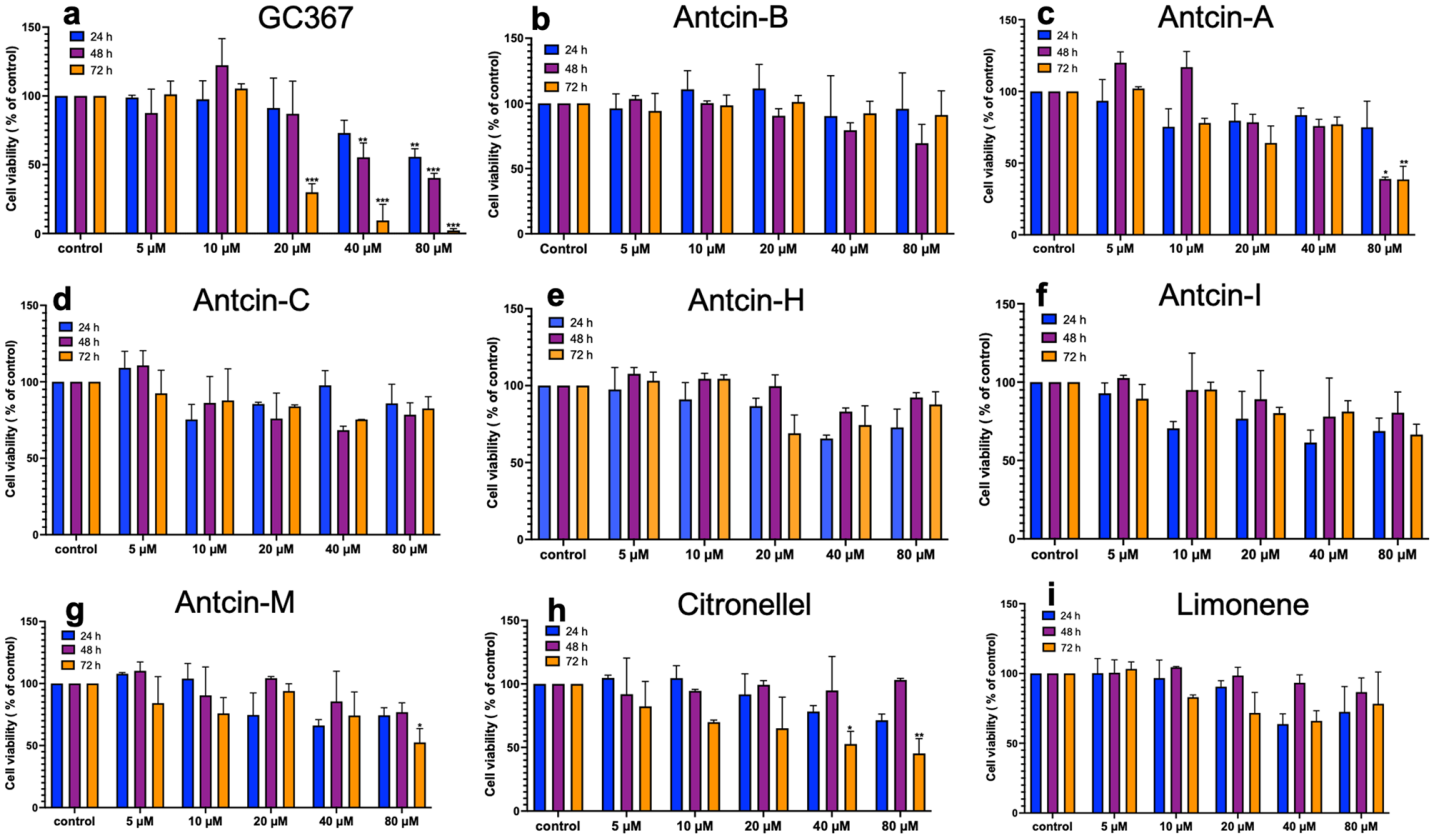
Cell viability effect of natural compounds on A549 cells. Cytotoxicity effects of (**a**) GC376, (**b**) antcin-A, (**c**) antcin-B, (**d**) antcin-C, (**e**) antcin-H, (**f**) antcin-I, (**g**) antcin-M, (**h**) citronellol, and (**i**) limonene. Antcin (A, B, C, H, I, and M), non-antcins (citronellol and limonene), and the positive control compound (GC376) on A549 cells. The cells were incubated with increasing concentrations of antcins, non-antcins, and GC376 (5–80 μM) for 24, 48, and 72 h. Cell toxicity was determined by the MTT colorimetric assay as described in the materials and methods. Values represent the mean ± S.D. of three independent experiments. Statistical significance was set at *p < .05, **p < .01, ***p < .001 compared to the treatment and control groups. Without asterisks, indicate statistically non-significant results.

Before delving into the SARS-CoV-2 3CL^Pro^ inhibitory effects of antcins, it is vital to assess the cytotoxic effects of antcins on A549 cells. To evaluate cytotoxicity, we treated A549 cells with various antcins (antcin-A, antcin-B, antcin-C, antcin-H, antcin-I, and antcin-M), non-antcin compounds (citronellol and Limonene), and GC376 at increasing concentrations (5–80 µM) for 24, 48, and 72 h under 5% CO2 at 37 °C. GC376 was developed as a pharmacological inhibitor of 3CL^Pro11^^11^ as a positive control compound. Based on the MTT assay, we found that treatment with GC376, a positive drug control, caused significant cytotoxicity over a concentration of 40 µM at 48 and 72 h, and 20 µM GC376 decreased cell viability to 30% after 72 h of incubation (Fig. 2a). Except antcin-A, other antcins did not exhibit significant cytotoxicity towards A549 cells even at a concentration of 80 µM for 24 h (Fig. 2b–g). At the same time, antcin-A and antcin-M exhibited significant cytotoxicity at a dose of 80 µM at 48 and 72 h. Likewise, citronellol did not induce cytotoxicity up to an amount of 20 µM, whereas 40 µM and 80 µM caused cytotoxicity at 72 h (Fig. 2h). Indeed, Limonene exhibited significant cytotoxicity neither in a concentration- nor time-dependent manner (Fig. 2i). These findings collectively suggest that antcins have lower cytotoxicity towards A549 cells compared to GC376. Furthermore, antcins appear to have no potential adverse effects on cells, making them suitable for drug screening and further investigation. Consequently, based on the MTT assay results, we selected all the above compounds for screening in the docking study and other experiments.

### Drug-likeness, physicochemical properties, toxicity risk assessment, and ADMET prediction of antcins

Drug discovery and development pose significant challenges and costs when targeting specific diseases involving multiple stages such as selection, target identification, validation, lead discovery, optimization, and preclinical and clinical trials^31^. Therefore, crucial factors such as chemical absorption, distribution, metabolism, excretion, and toxicity (ADMET) play a vital role in drug discovery and development^32^. In silico models can accurately predict ADMET properties based on specific parameters, aiding in identifying viable drug candidates for particular diseases. To evaluate the drug-likeness of the ligand's physicochemical properties and assess its toxicity risk, we employ the OSIRIS Property Explorer. This tool allows us to evaluate parameters such as solubility (ranging from 0 to − 6), TPSP (topological polar surface area ≤ 130 Å^2^), drug-likeness (scores with a positive value are likely to be an oral drug), drug score (0–1), and molecular weight (MW) lower than 500 g/mole. Based on our analysis, all the tested compounds exhibit moderate solubility, TPSA values of 130 Å^2^, and drug scores within a range of 0–1 (Table S1), which indicate favorable drug-likeness and low toxicity risks. Notably, this prediction aligns with the cytotoxicity of the tested compounds (Fig. 2). Next, we screened the ADMET properties of test compounds using AdmetSAR server2 [http://lmmd.ecust.edu.cn/admetsar2]^33^. In silico toxicity, testing of our hit compounds reveals insights into their pharmacokinetics and pharmacodynamics compared to the selected control drugs. Regarding absorption, Caco-2 permeability testing predicted that all compounds, including GC376, to be absorbed by the human intestine. The combinations were also expected to exhibit human oral availability, p-glycoprotein inhibition, and act as substrates. None of the compounds were deemed AMES mutagenic or carcinogenic and were anticipated to be non-toxic to liver cells. The predicted acute oral toxicity (kg/mol) is presented in Table S2. Two properties, plasma protein binding (PPB) and blood–brain barrier (BBB) permeability, were utilized to assess the compound’s distribution. All the compounds exhibited the potential to reach their target site at high to moderate doses, as their ability to bind to plasma proteins was predicted to be over 95% in most cases. However, GC376 (58.38%) and Limonene (86.38%) showed a lower likelihood of binding to plasma proteins, as indicated in Table S2. Moreover, all the compounds were predicted to function as substrates or inhibitors of various cytochrome enzymes, including CYP1A2, CYP2C19, CYP2C9, CYP2D6, and CYP3A4, as shown in Table S2. Additionally, the predicted acute oral toxicity (kg/mol) for the compounds is provided in Table S2.

### Antcin-B has the best binding affinity energy determined by molecular docking

**Figure 3:**
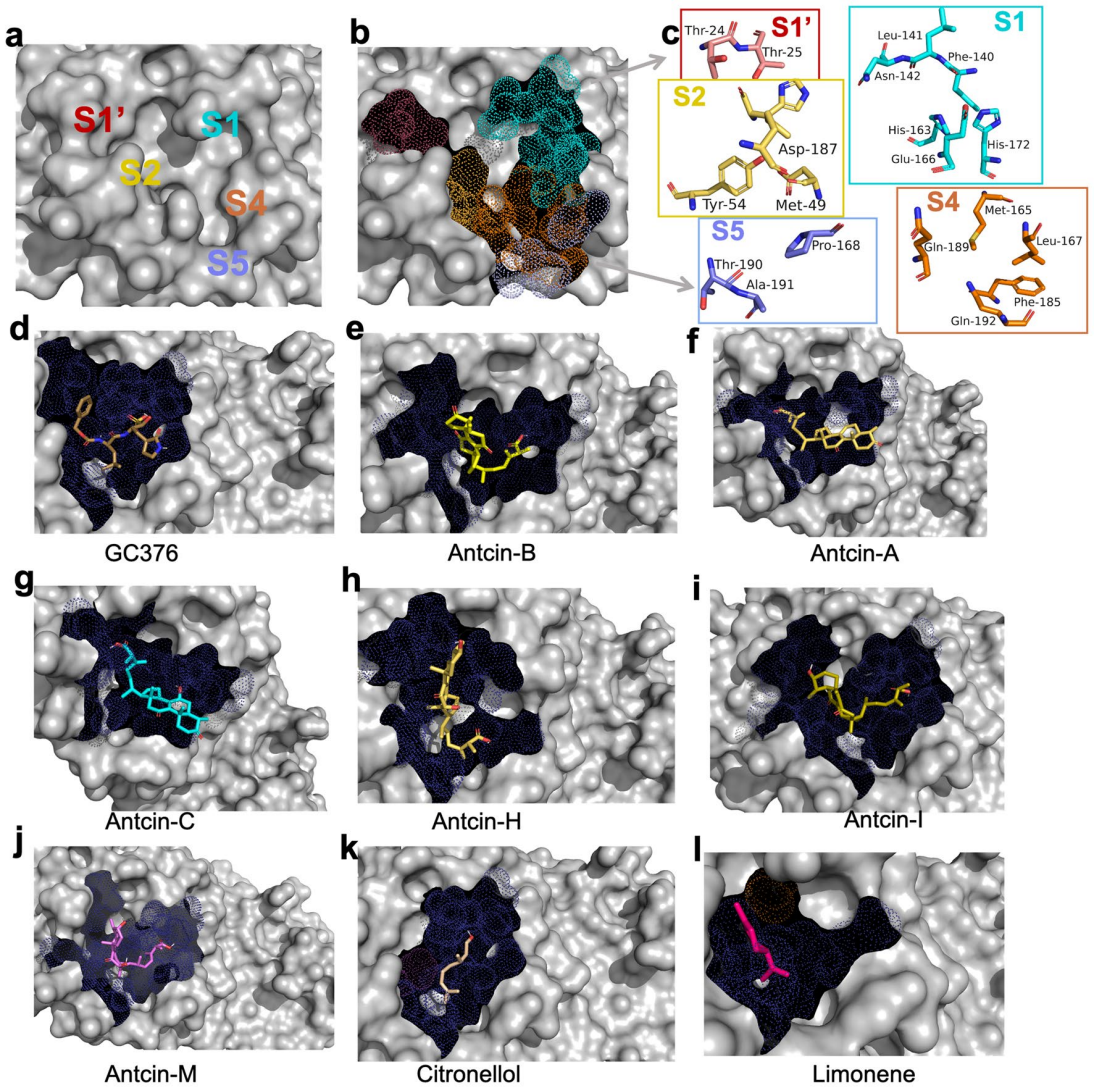
Structural analysis and potential inhibitors with SARS-CoV-2-3CL^Pro^ enzyme using molecular docking. (**a**) Cocrystal structures of SARS-CoV-2-3CL^Pro^ labeling substrate binding subsites (S1, S2, S4, S5, S1′). (**b**) Binding subsites of 3CL^Pro^ are presented in dot models, with S1, S1′, S2, S4, and S5 shown in different colors (maroon, cyan, yellow, orange, and purple), and (**c**) these five substrate binding subsites comprise the main catalytic site with amino acid residues and their structures presented. Since these are catalytic binding sites, (**d**–**l**) antcins, non-antcins, and the positive control GC376 were docked with the active site of 3CL^Pro^ protein using CB-Dock2 and Auto-Dock Vina module; UCSF-Chimera. The compounds bind to highly similar pockets of binding sites. The binding sites are presented with a dot model in deep blue. Ligands (compounds) binding with SARS-CoV-2-3CL^Pro^ are presented in stick models with different colors, as shown in (**d**–**l**).

With an understanding of the molecular catalytic pattern of SARS-CoV-2-3CL^Pro^, we obtained a complex structure (PDB code: 7LME)^34^. We uploaded 3CL^pro^ and ligand-processed structures using PyMOL, as explained in the Methods section, to facilitate the docking process. The detailed structure of 3CL^Pro^ and the catalytic binding site is shown in (Fig. S1a–d). Generally, SARS-CoV-2 3CL^Pro^ appears as a quasi-ellipsoid that possesses three separate domains (domain I, domain II, and domain III), which are connected by flexible loops (Fig. S1b, c) and a catalytic binding pocket with subsite binding clefts, including S1, S2, S1′, S4, and S5^35,36^ as shown in Fig. 3b. The docking was performed with antcins and non-antcin compounds and the positive control compound (GC376) with SARS-CoV-2-3CL^Pro^ using CB-Dock2, which is predicted to have more than an 85% success rate compared to FitDock, MTiAutoDock, SwissDock, and COACH-Dn^27^. After docking, the conformation with the least binding energy (BE) kcal/mol) was considered the most suitable docking pose of the 3CLPro for the ligand, as all tested compounds were bound within the catalytic binding pocket with the subsite binding cleft of the 3CL^pro^, as shown in Fig. 3d–l. Detailed 3-dimensional coordinates where the ligand bound to the protein as center size (x, y, z) and docking size (x, y, z) are presented in Table 1. Docking with 3CL^Pro^, all the antcins, including antcin-A, antcin-B, antcin-C, antcin-H, antcin-I, and antcin-M, produced BE high values of − 7.2, − 8.2, − 7.2, − 7.6, − 8, and − 8.1 kcal/mol, respectively. In contrast, citronellol and Limonene showed much higher BE values of − 4.2 and − 4.1 kcal/mol, respectively. Interestingly, GC376 produced a BE of − 7.1 kcal/mol, comparable with antcins, possibly due to all the antcins having a similar structure as shown in Fig. 1, and found these compounds bound with 3CL^pro^ in a similar pattern and at similar active sites, as shown in Table 1 and Fig. 3d–l. The binding site of 3CL^Pro^ is contacted by various residues, including Glu166, Leu141, Phe140, His172, His163, Asn142, Thr25, Thr24, Met165, Met49, Asp187, His41, Thr54, Leu167, Phe185, Gln192, Gln189, Met165 168, Ala191, and Thr190, located in subsites of 3CL^Pro^ such as S1, S1′, S2, S4, and S5, as shown in Fig. 3a–c^35,36^. These residues might explain the inhibitory activity of these compounds, as residues like Thr24 form a part of the catalytic site for the inhibition of 3CL^pro^^37^. However, antcin-B exhibits the best binding affinity energy, contacting 19 amino acids within the subsite binding cleft, including S1′-(Thr25 and Thr24), S1-(Glu166, Leu141, Phe140, Asn142, and His163), S2-(Met165, Met49, His41, S4-Gln189), which are mainly responsible for substrate binding^38^, whereas Cys44, Thr45, Ser46, Ser144, Cys145, and Arg188 are responsible for the enzyme dimeric structure of the 3CL^pro^ and not conventional catalytic residues for the ligands, which might be the reason for better affinity binding than other compounds as shown in Table 1. However, molecular docking relies on specific parameters for different software or webtools where the docked complexes must reproduce the original poses of the native ligands with precision. Therefore, the complex was redocked using the UCSF-Chimera interestingly revealed that antcin-B exhibited the best binding energy of − 9.3 kcal/mol, followed by antcin-H, antcin-M, antcin-I, antcin-A, antcin-C, GC376, citronellol, and Limonene with B.E. values of − 7.6, − 7.4, − 7.2, − 7.2, − 6.7, − 7.4, − 4, and − 4.1 kcal/mol, respectively that align with the CB-Dock2 binding presented in Table 1. Therefore, antcin-B exhibited the best binding energy among the test compounds, including the positive compound GC376. It highlights antcin-B as a promising candidate for further investigation, possibly contributing to their potential efficacy or interaction with the target residues via specific interaction.

**Table 1:** Results of binding affinity (Kcal/mol), cavity and docking size, and contacts residues from non-antcins and GC376, which were docked against the SARS-CoV-2 3CL^Pro^ protein using CB-dock2 and Auto-Dock Vina module; UCSF-Chimera.

PubChem CID	Compounds name	Kcal/mol (1)	Kcal/mol (2)	Cavity Volume (Å^3^)	Center (x, y, z)	Docking size (x, y, z)	Contact residues
44424392	Antcin-A	− 7.2	− 7.2	397	27, 0, 14	25, 25, 25	Thr25 His41 Val42 Cys44 Thr45 Ser46 Met49 Phe140 Leu141 Asn142 Cys145 His163 His164 Met165 Glu166 Arg188 Gln189
387397	Antcin-B	− 8.7	− 9.3	397	27, 0, 14	24, 24, 24	Thr24 Thr25 His41 Cys44 Thr45 Ser46 Met49 Phe140 Leu141 Asn142 Gly143 Ser144 Cys145 His163 His164 Met165 Glu166 Arg188 Gln189
10050412	Antcin-C	− 7.2	− 6.7	397	27, 0, 14	25, 25, 25	Thr25 Leu27 His41 Val42 Cys44 Thr45 Ser46met49 Phe140 Leu141 Asn142 Gly143 Ser144 Cys145 His163 His164 Met165 Glu166 Arg188 Gln189
68150384	Antcin-H	− 7.6	− 7.6	397	27, 0, 14	25, 25, 25	Thr24 Thr25 Thr26 Leu27 His41 Ser46 Met49 Asn142 Gly143 Cys145 His164 Met165 Glu166 Leu167 Arg188 Gln189
Not Yet	Antcin-M	− 8	− 7.4	397	27, 0, 14	23, 23, 23	Thr25 Thr26 His41 Cys44 Thr45 Ser46 Met49 Phe140 Leu141 Asn142 Ser144 Cys145 His163 His164 Met165 Glu166 His172 Gln189
Not Yet	Antcin-I	− 8.1	− 7.2	397	27, 0, 14	23, 23, 23	Thr24 Thr25 Thr26 His41 Cys44 Thr45 Ser46 Met49 Phe140 Leu141 Asn142 Ser144 Cys145 His163 His164 Met165 Glu166 His172 Gln189
8842	Citronellol	− 4.1	− 4	397	27, 0, 14	19, 19, 19	His41 Met49 Phe140 Leu141 Asn142 Gly143Ser144 Cys145 His163 His164 Met165 Glu166 Asp187 Arg188 Gln189
22311	Limonene	− 4.2	− 4.1	397	27, 0, 14	17, 17, 17	His41 Met49 Asn142 His164 Met165 Glu166 Val186 Asp187 Arg188 Gln189
71481119	GC376	− 7.1	− 7.4	397	27, 0, 14	24, 24, 24	Thr25 Thr26 Leu27 His41 Met49 Phe140 Leu141 Asn142 Gly143 Ser144 Cys145 His163 His164 Met165 Glu166 Leu167 His172 Arg188 Gln189

Antcin-I and antcin-M may have yet to be submitted to PubChem as the chemical compound identification number was not found. Kcal/mol (1) is the binding affinity energy score for which CB-Dock2 and Kcal/mol (2) were determined using the Dock Vina module; UCSF-Chimera. CB-Dock2 produces the contact residues but is not analyzed using other programs. Active residues like Cys145 and His41 are highlighted in red, but Limonene has not come into contact with Cys145. Antcin-B has the lowest binding affinity energy in CB-Dock2 and UCSF-Chimera, which explains why antcin-B completely inhibited 3CL^Pro^ activity. For the center size (x, y, z) and docking size (x, y, z), we employed CB-Dock2 for the docking, which provides the three-dimensional coordinates of the protein region in two forms known as center size (x, y, z), which defines the central point within the binding site of the receptor where the ligand is positioned and docked. Docking size (x, y, z) is the coordinate of the ligand bound to the protein. Thus, our result, three-dimensional coordination with protein where the ligand is bound, is similar to antcins' structures and targets the same catalytic site of the protein. The smaller docking size is better for binding to the target residues. Details of binding interactions of antcins (A, B, C, H, I, and M), non-antcins (citronellol and Limonene), and GC376 docked into the active site of SARS-CoV-2-3CL^Pro^ and forming a ligand–protein complex.

### Antcin-B inhibits 3CL^Pro^ activity via hydrophobic interaction, hydrogen bonding, and a salt bridge

**Figure 4:**
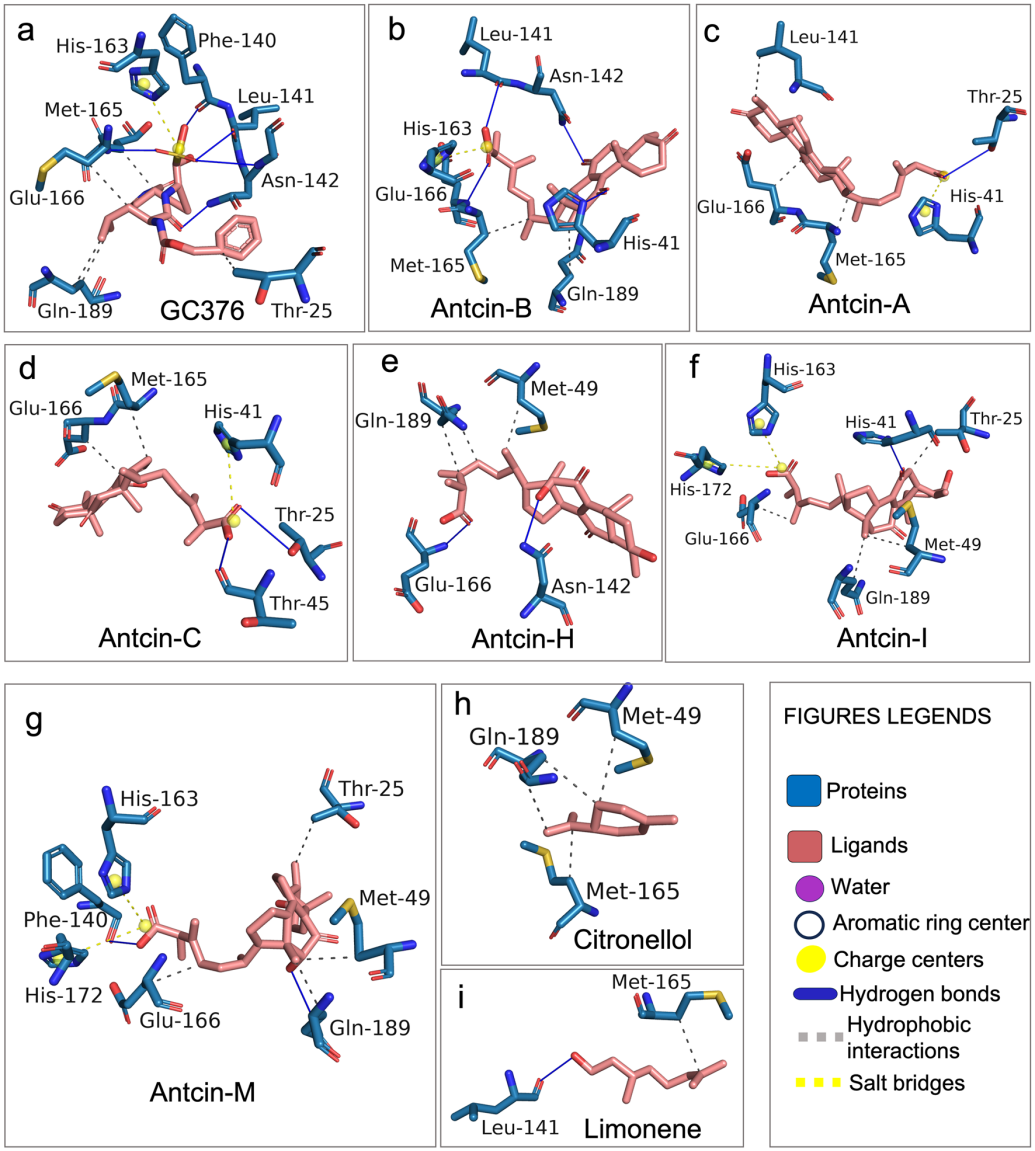
Detailed visualization of the interaction between the native ligand and residues of SARS-CoV-2-3CL^Pro^ using PLIP from the docking results. Antcins, non-antcins, and the positive control GC376 were docked with the active site of the 3CL^Pro^ protein using CB-Dock2 and Auto-Dock Vina module; UCSF-Chimera, exhibiting similar binding pockets but different binding affinity energy as shown in Table 2. The detailed chemical interaction of the above compounds with 3CL^Pro^ is performed by the protein–ligand interaction profiler (PLIP), allowing detection and visualization of relevant interactions from docking results. Ligands interacting with the amino acids of 3CL^Pro^ are presented in stick models with the same color as a flamingo for ligands. The ligands interact with residues of 3CL^Pro^ via hydrogen bonds, hydrophobic interactions, and salt bridges, as shown by PLIP. (**a**) GC376, (**b**) antcin-A, (**c**) antcin-B, (**d**) antcin-C, (**e**) antcin-H, (**f**) antcin-I, (**g**) antcin-M, (**h**) citronellol, and (**i**) limonene interact with various 3CL^Pro^ residues represented in stick models. Interactions in the blue line indicate hydrogen bonds between drugs and amino acids, the grey dotted line represents hydrophobic interactions, and the yellow dotted line indicates the salt bridge in PLIP results.es legends. Residue contacts represent the blue color, and the red dot represents Oxygen.

We employed PLIP^29^ and Protein*Plus*^39^ to determine the types of chemical interaction obtained from the ligand–protein complex. These ligands were found to bind to the catalytic site of the 3CL^Pro^ (Table 1, Fig. 3a–l). Even though the ligands fit similar cavities, their interaction with amino acids varied when analyzed with PLIP (Table 2). As given in the positive control, GC376 displayed interactions with 10 amino acids that are part of the subsites of 3CL^Pro^. These interactions including hydrogen bonds and hydrophobic interactions, were reported by Fu et al. and Vuong et al.^11,40^, except for forming the salt bridge with His163 (Fig. 4a, Table 2), consistent with previous research^41^. However, the absence of a salt bridge formation with His163 distinguishes this study's findings from those of previous works. Interestingly, antcin-B exhibited the best BE and interaction with 6 amino acids resembling GC376, which include hydrophobic interaction with Glu169 and Gln189, while the other four amino acids (His41, Leu141, Asn142, and Glu166) engaged in hydrogen bonding. Moreover, it forms a salt bridge, specifically with His163, which may indicate a strong interaction between antcin-B and the binding site (Fig. 4b, Table 2). This might explain why antcin-B displayed the highest docking score and more hydrogen bond interactions^42^. Because antcin-A shared the same binding cavity with antcin-B, antcin-A engaged in hydrophobic interaction with Leu 141, Met165, and Glu166, which formed a hydrogen bond with Thr25 and a salt bridge with His41 while not with His163 (Fig. 4c, Table 2). Antcin-C only contacted Met165 and Glu166 via hydrophobic interaction, interacted with Thr25 and Thr45 via hydrogen bonding, and formed a salt bridge with His41 and not with His163 (Fig. 4d, Table 2). Thus, creating a salt bridge with His163 suggests a specific and compelling interaction at that site that likely contributes significantly to its favorable docking score. As shown in Fig. 4f, g and Table 2, both antcin-I and M produce a salt bridge with His163 and His172, whereas antcin-H makes neither contact with His41 nor His163, His172 through the salt bridge (Fig. 4e; Table 2). Citronellol interacted with His41 through hydrogen bonding, whereas Limonene engaged in hydrophobic interactions with three amino acids, Met49, Met165, and Gln189, respectively (Fig. 4h, i), with predictably lower docking scores. The ligand–protein interactions were further validated using Protein*Plus*, which offers detailed 2D ligand–protein interaction, and PoseView, which provides hydrogen and hydrophobic interaction^30^. The complex interaction is provided in Fig. S3a–h. However, it doesn’t demonstrate salt bridge formation because it focuses on polar interactions like hydrogen and hydrophobicity^39^. These results indicate that the hydrogen bond, Hydrophobic interaction, and salt bridge occur at residues such as Glu169 and Gln189, His41, Leu141, Asn142, Glu166, and His163 with antcin-B are essential factors in increasing the binding affinity energy in the docking like previous studies^43^. Antcin-B's interaction with His163 through the salt bridge seems to be a key contributor to its strong binding affinity, underscoring the complexity of the binding process and the influence of distinct molecular interactions on the overall docking score, as salt bridges with His163, which were absent in the antcins (A, C, and H), citronellol, and Limonene, might not inhibit 3CL^Pro^ activity^44^. Interestingly, Antcin-I and M formed a salt bridge with His163 and His172 and a similar docking score with antcin-B that indicates either His163 or His172 is a critical binding residue to increase the docking. However, further experiments are needed to validate the results.

**Table 2:** Details of binding interactions of antcins (A, B, C, H, I, and M), non-antcins (citronellol and Limonene), and GC376 docked into the active site of SARS-CoV-2-3CL^Pro^ and forming a ligand–protein complex.

Ligand–protein complex	Interaction	Residues (AA)	Bond length (Å)
Antcin-A-SARS-CoV2-3CL^Pro^	Hydrophobic	Leu141, Me165, Glu166	3.89, 3.93, 3.7
	Hydrogen bonds	Thr25	3.76
	Salt Bridges	His41	4.8
Antcin-B-SARS-CoV2-3CL^Pro^	Hydrophobic	Met165, Gln189A	3.94, 3.95
	Hydrogen bonds	His 41, Leu141, Asn142 Glu166	2.71, 2.77, 2.67 3.1
	Salt Bridges	His163	4.07
Antcin-C-SARS-CoV2-3CL^Pro^	Hydrophobic	Met165, Glu166	3.8, 3.76
	Hydrogen bonds	Thr25, Thr45	3.78, 3.51
	Salt Bridges	His41	4.69
Antcin-H-SARS-CoV2-3CL^Pro^	Hydrophobic	Met49, Gln189	3.65, 3.79
	Hydrogen bonds	Asn142, Glu166	3.15, 2.47
	Salt Bridges	×	×
Antcin-I-SARS-CoV2-3CL^Pro^	Hydrophobic	Thr25, Met49, Glu166, Gln189	3.49, 3.82, 3.56, 3.91
	Hydrogen bonds	His41	2.71
	Salt Bridges	His163, His172	4.46, 5.45
Antcin-M-SARS-CoV2-3CL^Pro^	Hydrophobic	THr25, Met49, Glu166, Gln189	3.83, 3.79, 3.59, 3.6
	Hydrogen bonds	Phe140, Gln189	2.47, 3.275
	Salt Bridges	His163, His172	4.42, 5.45
Citronellol-SARS-CoV2-3CL^Pro^	Hydrophobic	Met165	3.85
	Hydrogen bonds	Leu141	2.62
	Salt Bridges	×	×
	Interaction	Residues (AA)	Bond length (Å)
Limonene-SARS-CoV2-3CL^Pro^	Hydrophobic	Met49, Met165, Gln189	3.87, 3.78, 3.87
	Hydrogen bonds	×	×
	Salt Bridges	×	×
GC376-SARS-CoV2-3CL^Pro^	Hydrophobic	Thr25, Met165, Glu166, Gln189	3.67, 3.78, 3.62, 3.92
	Hydrogen bonds	Phe140, Leu141, Asn142, Gly143, Glu166	3.42, 2.94, 2.915, 3.56, 2.975
	Salt Bridges	His163	4.76

Interaction details the ligand–protein complex was performed by using the PLIP. Three types of interaction were observed: hydrophobic interaction, hydrogen bonds, and salt bridges. Residues (A.A.), Amino acid, and Bond length are measured in the (**Å**). X represents the absence of the salt bridge bond formation between the ligand and target protein.

### Antcin-B significantly reduced SARS-CoV-2 3CL^Pro^ enzymatic activity in vitro

**Figure 5:**
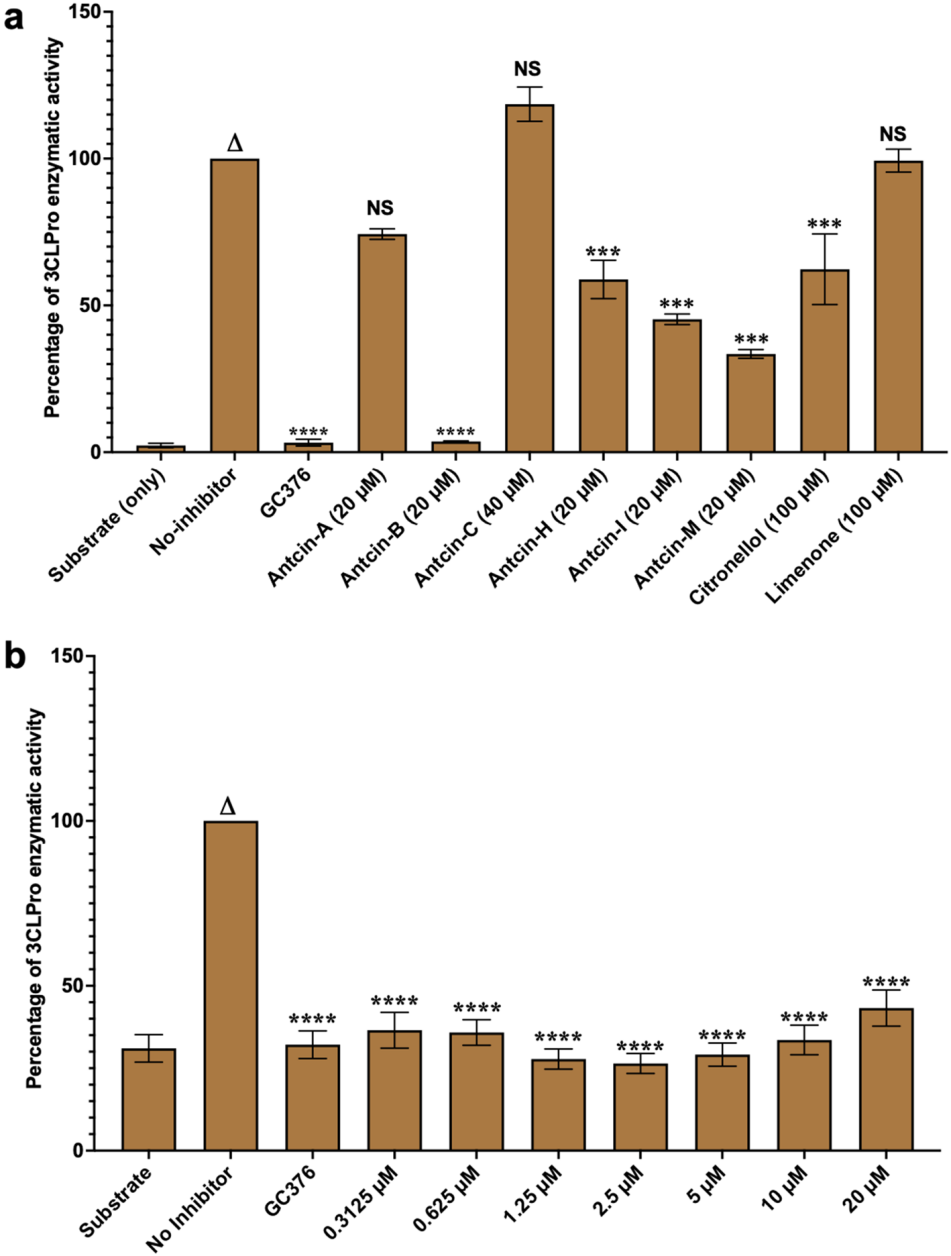
Antcin-B completely inhibits SARS-CoV-2 3CLPro like GC376 in enzymatic assay. Antcins (A, B, C, H, I, and M), non-antcins (citronellol and Limonene), and the positive control GC376 protease inhibitors were screened for their inhibitory activity against SARS-CoV-2 3CLPro enzyme, as described in the Methods section. The percentage (%) of inhibition of 3CL^Pro^ activity was calculated as the ratio of activity in the presence of the inhibitor to the total activity, considering the background. Blank values were subtracted from all the readings before calculating the percent activity. A representation of three individual experiments with triplicate values is presented graphically (n = 3). One-way ANOVA with Dunnett’s multiple comparison posthoc tests was used to calculate the statistical significance. Statistical significance was set at **p < .05, ***p < .001, ****p < .0001 compared to the treatment group versus the control group (no inhibitors). Without asterisks, indicate statistically non-significant (ns) results.

An in vitro enzymatic inhibitory assay using commercially available assay kits was performed to validate the results obtained from molecular docking. 3CL^Pro^ enzyme inhibitory effects of antcins (A, B, H, I, and M) were screened at a 20 µM concentration, as indicated by the docking results, showing potent inhibition. However, antcin-C, citronellol, and Limonene showed weaker interactions during molecular docking. Therefore, higher concentrations of 40 µM, 100 µM, and 100 µM, respectively, were used. GC376 (100 nM) was used as a positive drug control per manufacturer guidelines. The 3CL^Pro^ enzyme inhibitory assay revealed that compared with the control group (no inhibitor), treatment with GC376 as a positive control, antcin-A, antcin-B, antcin-H, antcin-I, antcin-M, and citronellol significantly inhibited 3CL^Pro^ activity by 96.72%, 25.7%, 96.39%, 41.17%, 54.74%, 66.54%, and 37.7% respectively (Fig. 5a). Whereas, antcin-C (− 18.5%) and Limonene (0.7%) failed to inhibit 3CLPro activity (Fig. 5a). Interestingly, antcin-B inhibited enzymatic activity with 96.39%. In contrast, antcin-I and M inhibited considerably with 54.74% and 66.54%, which is not as good as the positive control and antcin-B (Fig. 5a). This finding shows that antcin-B has the highest inhibitory activity of the 3CL^pro^ among tested compounds. It also explains the reason consistent with antcin-B has the best binding affinity energy and strong interaction with target residues like His41, Leu141, Asn142, Glu166, and His163 during docking. The similarity in inhibition percentages between antcin-B and GC376 highlights the potential of antcin-B as a novel inhibitor, possibly comparable to established compounds. Thus, only the Antcin-B has been selected for further dose-dependent study to determine the optimal concentration to inhibit maximum 3CL^Pro^ enzymatic activity. A dose-dependent analysis with increasing concentrations (0.3125, 0.625, 1.25, 2.5, 5, 10, and 20 μM) of antcin-B was conducted and used GC376, a positive control (100 ng/mL) based on the method section. The results revealed that antcin-B exhibited the maximum inhibitory activity against 3CL^Pro^ at a concentration of 2.25 μM, surpassing the effectiveness of the positive drug control GC376 (Fig. 5b), which indicates antcin-B could be a potentially promising natural product that blocks the activity of 3CL^pro^, surpassing pharmacological inhibitors like GC376, with low toxicity and strong interaction with catalytic residues.

### 100 nanoseconds (ns) molecular dynamics (MD) simulation shows that antcin-B has the highest stable interaction with 3CL^Pro^

To validate the experimental results, 100 ns molecular dynamics (MD) simulations were conducted for all ligand–protein complexes, where the docking results were used as the initial states for the simulations. A focus was placed on the protein–ligand interactions formed during the MD simulations to investigate the differences in binding activity to discover interactions between the potent and the weak compounds. An interaction was only taken into account if it was present for at least 15% of the simulation duration. Supplementary data of the MD simulations is included in Figs. S4 and S5.

**Figure 6:**
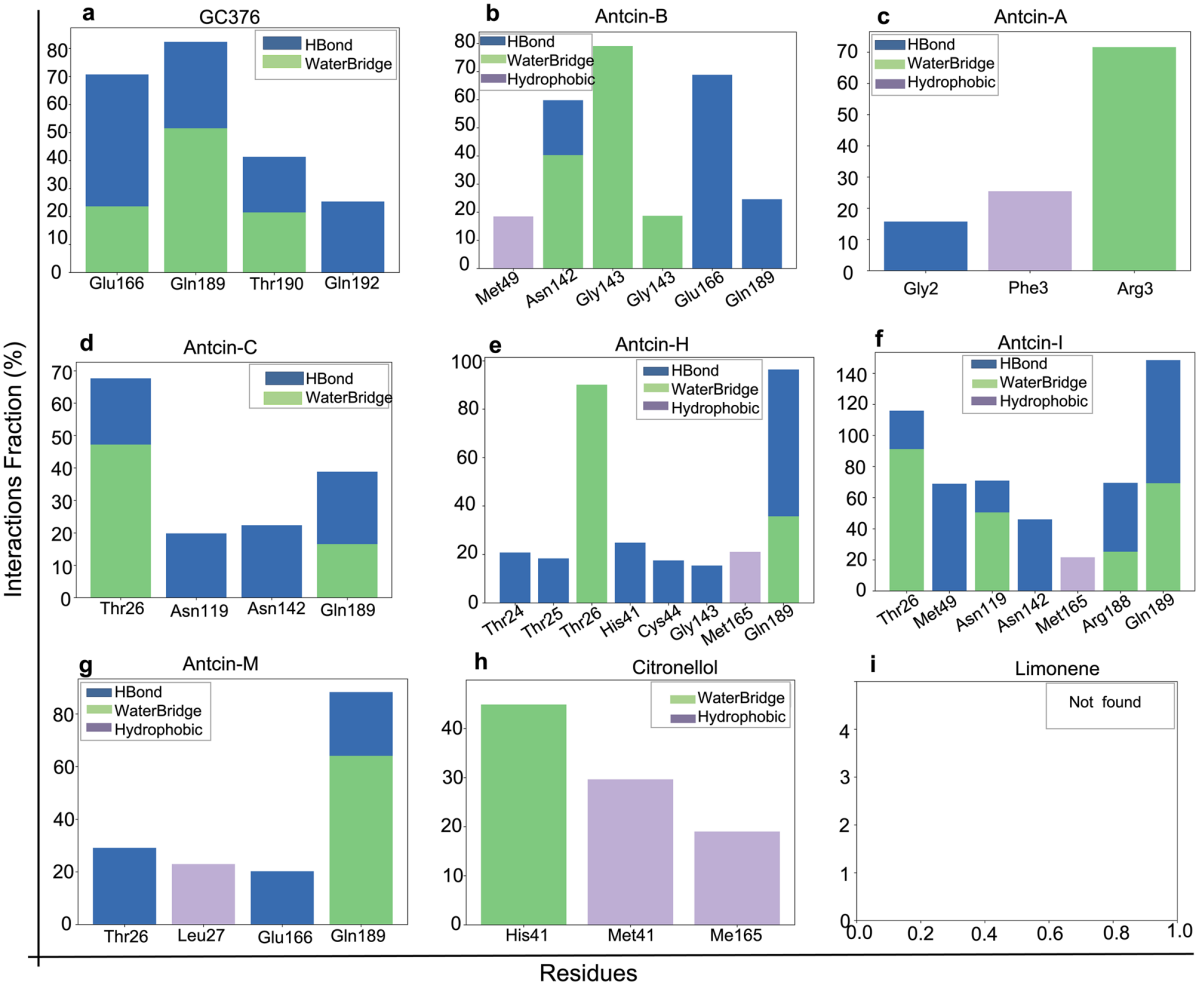
Protein–ligand interactions histograms from Molecular dynamics (MD) simulations (100 ns) of antcins (A, B, C, H, I, and M), non-antcins (citronellol and Limonene), and the positive control compound (GC376) with SARS-CoV-2-3CL^Pro^ (PDB ID; 7LME). Protein interactions with the ligand were monitored throughout the simulation, and the interaction types were classified as Hydrogen Bonds, Hydrophobic, and Water Bridge, represented by blue, green, and lavender colors, respectively. The Y-axis represents the interaction fraction (the % of the time the ligand formed an interaction with a certain protein residue). On the x-axis, the specific protein residues are plotted. Residues interacting for less than 15% of the simulation time were omitted for clarity. The protein–ligand mapping shows the normalized stacked bar chart depicting the intermolecular interaction simulation time fraction between 3CL^Pro^ with (**a**) GC376, (**b**) antcin-A, (**c**) antcin-B, (**d**) antcin-C, (**e**) antcin-H, (**f**) antcin-I, (**g**) antcin-M, (**h**) citronellol, and (**i**) limonene during the 100 ns MD simulation interval. Values of interaction fractions > 1.0 are possible, as some residues create multiple interactions of a similar subtype. Alternate figures displaying similar information are included in the supplementary data (Fig. S4a–i).

The control compound GC376 displayed a notable interaction with Glu166, where a hydrogen bond and a water bridge were formed and persisted for approximately 70% of the simulation time. GC376 demonstrated a sustained interaction with Gln189 and, to a lesser extent, with Thr190 and Gln192 (Fig. 6a; Fig. S5a–i). As expected, antcin-B displayed significant interactions, which include hydrogen bonds with Asn142 and Gly143, as well as a consistent water bridge with Glu166, which occurred for approximately 40%, 20%, and 70% of the simulation duration, respectively (Fig. 6b; Fig. S5b). The other antcin compounds displayed weaker interactions, reflected in their protein–ligand interactions. Antcin-A formed only two hydrogen bonds with Arg4, each persisting for approximately 35% of the simulation time. A hydrophobic interaction with Phe3 was observed for slightly over 20% of the simulation time (Fig. 6c; Fig. S5c). Antcin-C, which exhibited higher potency than antcin-A, formed a hydrogen bond with Thr26 for approximately 45% of the simulation time. Short-term water bridges were observed with Asn119, Asn142, and Gln189, each present for less than 20% of the simulation time (Fig. 6d; Fig. S5d). However, antcin-H, antcin-I, and antcin-M demonstrated longer-lasting interactions compared to antcin-A and antcin-C. Antcin-H established hydrogen bonds with Thr26 (approximately 90% of the time) and formed a water bridge with Gln189 for around half of the simulation time (Fig. 6e; Fig. S5e). Antcin-I exhibited hydrogen bonds with Thr26 (~ 90%), Asn119 (~ 50%), and Gln189 (~ 60%). Additionally, water bridges were formed with Met49 (~ 70%), Asn142 (~ 50%), Arg188 (~ 40%), and Gln189 (~ 70%) (Fig. 6f; Fig. S5f.). Antcin-M displayed weaker interactions, the most notable being a hydrogen bond with Gln189, present for 60% of the simulation time. Water bridges were also observed with Thr26 and Gln189 for 30% and 20% of the time, respectively (Fig. 6g; Fig. S5g). Citronellol formed a hydrogen bond with His41 for approximately 45% of the simulation time. In comparison, hydrophobic interactions with Met49 and Met165 were observed for around 30% and 20% of the simulation time, respectively (Fig. 6h; Fig. S5h), which did not significantly. Limonene did not exhibit significant interactions throughout the simulation (Fig. 6i; Fig. S5i). The observed patterns of protein–ligand interactions in the MD simulations can help explain the obtained experimental results, providing partial insights into the compounds with the lowest activity, such as citronellol and Limonene, were unstable during the simulation, which was reflected in the fact that they didn't form any significant interactions with the protein. More importantly, antcin-B and the control compound GC376 included consistent, long-lasting water bridges with Glu166 that align with docking and experimental result persistence.

## Discussion

SARS-CoV-2-3CL^Pro^ is vital in viral replication and has exhibited significant conservation across various coronaviruses for targeting drug discovery. Several studies have used natural and synthetic compounds targeting SARS-CoV-2-3CL^Pro^, covalent compounds derived from peptidic scaffolds with an electrophile that reacts with the catalytic cysteine Cys145 and His41^10–15^. One of the well-known synthetic compounds is GC376, which can inhibit 3CL^Pro^ activity^45^. However, the role of the natural derivative compounds from *T. camphoratus* in inhibiting 3CL^Pro^ activity still needs to be discovered. Initially, the assessment of cytotoxicity test results plays a critical role in determining the potential of a small molecule in the drug discovery pipeline^46^. This study demonstrated that GC376 caused a reduction in cell viability exceeding 30% at concentrations of 40 and 80 μM after 48 h of incubation. In contrast, antcins, citronellol, and Limonene exhibited less cytotoxicity, implying that these compounds are more likely to be well-tolerated in host organisms. This implies that antcins derived from *T. camphoratus* have demonstrated fewer harmful effects or adverse reactions within cellular environments than GC376.

While the reason for the inhibitory effect of the tested compounds towards 3CL^pro^ is not clearly understood, the strength of hydrogen bonding interactions between ligands and 3CL^pro^ or forming the salt bridge with His163 along hydrogen bonds may explain differential inhibitory effects^47,48^. We have performed the MD simulation, post-analysis process, and enzymatic assay to understand the chemical mechanism. It was discerned that antcin-B exhibited the highest docking score, closely paralleling the scores attained by Antcin-I and M. Notably, antcin-B engaged with specific residues, including Phe140, Leu141, Asn142, Glu166, His163, and His172, derived from individual monomers of the 3CL^pro^ complex^49^ as in a manner reminiscent of GC376 that entailed the formation of a salt bridge with His163 and a more pronounced engagement of hydrogen bond interactions with Leu141, Asn142, Glu166, His163. This interaction has been shown to curtail viral replication effectively^40^ by fostering a network of interactions that includes residues His41, His164, and Asp187, thereby preserving essential polar contact^50^. In contrast, other compounds failed to form hydrogen bonds with these residues. Intriguingly, Antcin-B can inhibit more than 96% (almost wholly) of 3CL^pro^ activity in our enzymatic assay at 20 µM, which is comparable to the inhibition achieved by the GC376, which is a general commercial inhibitor of the 3CL^pro^^11,45^. Despite higher docking scores and interaction of antcin-I and M similarities with antcin-B with His163 and His172 via the salt bridge, antcin-I and M inhibited only with 54.74% and 66.54%, respectively, at 20 µM that did not demonstrate a commensurate level of significant inhibition of the 3CL^Pro^ as antcin-B and positive control. It is reported that mutation at His163 completely inactivates the enzymatic activity of 3CL^pro^, whereas modification at His172 still retains 26% of the enzymatic activity of 3CL^Pro^^44^. Thus, it suggests that the high docking score of antcin-B, along with its strong interaction only with His163 via the salt bridge, the multiple interactions Leu141, Asn142, and Glu166 through hydrogen bonds offer becomes a specific binding mode, which might be the reason behind its inhibitory solid activity against 3CL^Pro^ but forming salt bridges with both His163 and His172 like Antcin-I and M becoming a more versatile interaction pattern which might imply a broader binding mechanism that contributes to their moderate docking scores and potentially less potent inhibitory activity.

In addition, His-41 residues are essential residues in the binding site, which is part of the catalytic dyad His41, necessary for the proteolytic activity of 3CL^Pro^^51^. Forming a salt bridge with antcin-A and C did not significantly increase the docking score and had no potential inhibitory effect on 3CL^Pro^ activity. Thus, this study mainly signifies that only antcin-B, a phytosterol-like compound from *T. camphoratus*, inhibits the 3CL^Pro^ enzymatic activity by binding with the His163 residues through the salt bridge and hydrogen bond with Leu141, Asn142, and Glu166, highlighting the complexity and precision of its binding mode, which could lead to its high inhibitory potency. These similarities to previous studies, including baicalin^52^, theaflavin-3-O-gallate^53^, and nelfinavir^54^. MD simulation observed that antcin-B and the control compound GC376 formed consistent, long-lasting water bridges with Glu166, whereas these were not found with His163 via the salt bridge. Antcin-A and Antcin-C only showed less stable binding. Antcin-H and Antcin-I showed moderate 3CL^Pro^ inhibition, and in these simulations, they showed a lot more interactions.

The limitation of this study, docking scores, and MD simulation provide insights into binding affinity and stability interaction that may not entirely correlate with actual enzymatic inhibition, which further necessitates in vivo experimental and preclinical data due to the powerful invasion ability of SARS-CoV-2. Also, this study does not delve into the precise mechanisms behind the differing inhibitory effects of compounds antcin-I and M compared to antcin-B, despite having similar docking scores and binding with His163 and His172 or with Glu166 via water bridge. Furthermore, experimental studies, such as structural analyses and in vivo testing, would be crucial to validate these findings and determine their therapeutic potential.

In conclusion, this study successfully employed in silico and in vitro experiments to identify antcin-B as a potential inhibitor of SARS-CoV-2 3CL^pro^ which are derivatives from *Taiwanofungus camphoratus.* This study highlights possible chemical mechanisms by which antcin-B could become an important natural product for combating COVID-19 through binding at the S1 subunit via the salt bridge and hydrogen bonding interaction. Thus, further research is worth pursuing to understand the detailed mechanism and conduct in vivo testing for actual drug development for the inhibition of 3CL^pro^.

## Materials and methods

### Chemicals

GC376 (≥ 98%) (Cat # T5188) was purchased from TargetMol (Washington Street, Wellesley Hills, MA, USA). High glucose Dulbecco's Modified Eagle's Medium (DMEM)/high glucose containing 4 mM L-Glutamine, 4.5 g/L glucose (Cat # AE29444172) without sodium pyruvate, penicillin–streptomycin antibiotic solutions, and fetal bovine serum (FBS) were obtained from Hyclone/G.E. Healthcare Life Sciences (Logan, UT, USA). Antcins (A, B, C, H, I, and M) were isolated from fruiting bodies of *T. camphoratus* and *A. salmonea*, as described previously^16,55^, and purchased from the R&D Center of Taiwan Leader Biotechnology Corp. (Taichung, Taiwan) and purified using HPLC and FT-NMR analysis. GC-grade citronellol (Cat # C0370) and Limonene (Cat # L0132) were obtained from Tokyo Chemical Industry Co., Ltd (Toshima, Tokyo, Japan).

### Method for purification of the antcins

Fruiting bodies of *T. camphoratus* were obtained from the R&D Center of Taiwan Leader Biotechnology Corp (Taichung, Taiwan). The oven-dried fruiting bodies of *T. camphoratus* (50 g) were extracted with methanol (3 × 2 L) at room temperature (7 days each). The combined extract was evaporated under reduced pressure to afford a brown residue, suspended in H_2_O (1 L), and then extracted sequentially with EtOAc and n-BuOH (3 × 1 L). The EtOAc fraction (10.8 g) was subjected to silica gel chromatography (60 × 3.5 cm) using a stepwise gradient mixture of n-hexane and EtOAc as an eluent. Fraction 5 was purified through a silica gel column (2 × 45 cm) and eluted with CH_2_Cl_2_–EtOAc (30:1 to 0:1) to obtain 6 fractions (each about 300 mL), 5A–F. Fraction 5C was applied to semi-preparative HPLC eluted with CH_2_Cl_2_–acetone (40:1) to yield antcin-A (5.1 mg). Fraction 6 was further chromatographed on a silica gel column (2 × 45 cm), eluted with CH2Cl2–EtOAc (15:1 to 0:1) to resolve into 5 fractions (each about 350 mL), 6A–E. Fraction 6B was subjected to semi-preparative HPLC eluted with CH_2_Cl_2_–acetone (20:1) to yield antcin-B (9.5 mg). Fraction 6C was also subjected to semi-preparative HPLC eluted with CH_2_Cl_2_–acetone (15:1) to yield antcin-C (4.5 mg). Fraction 7 was further chromatographed on a silica gel column (2 × 45 cm) and eluted with CH_2_Cl_2_–methanol (50:1) to afford 7 fractions (each about 300 mL), 7A–G. Fraction 7C was subjected to semi-preparative HPLC eluted with CH_2_Cl_2_–acetone (9:1) to yield antcin-H (6.5 mg). Fraction 9 was further purified through a silica gel column (2 × 45 cm) and eluted with CH_2_Cl_2_–MeOH (15:1) to obtain 6 fractions (each about 300 mL), 9A–F. Fraction 9E was subjected to semi-preparative HPLC eluted with CH_2_Cl_2_–isopropanol (9:1) to yield antcin-K (4.1 mg). The five purified antcins in a mixture of two epimers at C25 were confirmed by their 1H-NMR, 13C-NMR, and HR-EI-MSN spectra. The structures of the antcins are described in Fig. 1. The purity of all antcins is higher than 99%, analyzed by HPLC and NMR spectrum^16,55^.

### Cytotoxicity assay and cell treatment

To investigate the cytotoxicity of GC376 and different antcins (A, B, C, H, I, and M), citronellol, and limonene were tested on A459 cells. Human lung cancer cell A549 was obtained from the bioresources collection and research center (BCRC, Hsinchu, Taiwan) at 4 passages of the cell lines. The culture was maintained for subculture under 5% CO_2_ at 37 °C for 3 days. Cells were seeded at a 1 × 10^6^ cells/mL per 10 cm dish density at eight passages of the cells. Under culture conditions, cells were allowed to reach approximately 90% confluency after seeding. Then, A549 cells were seeded in 96-well plates with 5000 cells per well in 200 μL of complete cell culture media, followed by incubation at 37 °C (5% CO_2_) overnight for cell adhesion on the plates. The cells were then treated with various doses of antcins (5–80 μM), GC376 (5–80 μM), citronellol (5–80 μM), and limonene (5–80 μM) for 24, 48, and 72 h. At the end of the experiment, 100 μL of 3-(4,5-dimethylthiazol-2-yl)-2,5-diphenyltetrazolium bromide (MTT), 0.5 mg/mL with media was added to each well, and the plate was incubated for 4 h. Excess MTT was then aspirated. The formazan crystals formed were dissolved in 100 μL of dimethyl sulfoxide (DMSO). The absorbance, proportional to cell viability, was measured at 570 nm using a microplate reader (μQuant, Biotek Instruments, Winooski, VT, USA). The percentage of cell viability (%) was calculated as (A570 of treated cells/A570 of untreated cells) × 100.

### Software and program for protein and ligand interaction

For the computational study, the software and websites used to execute this in silico study were as follows: Python Prescription 0.8 (PyRx) was used for optimizing ligands and molecular docking using the Auto-Dock Vina module; UCSF-Chimera (version 1.16), and CB-Dock2^27^ [https://cadd.labshare.cn/cb-dock2] was used for website docking. PyMOL molecular graphic system GL_VERSION: 2.5.4 was used for input data preparation, visualization, and processing data. Proteins*Plus* [https://proteins.plus] webtool tools are developed in the computational molecular design group (AMD) headed by Matthias Rareys, which is mainly life scientists working with protein structures that analyze post-docking results^56^ and Protein–Ligand Interaction Profiler (PLIP) [https://plip-tool.biotec.tu-dresden.de/plip-web/plip/index] for identifying the non-covalent interactions between biological macromolecules and their ligands^29^. All software was run on a personal computer, Apple M1 chip 8-core CPU, and 256 GB hard disk.

### Preparation of protein and ligands for docking

The crystal structure of the SARS-CoV-2-3CL^Pro^ in complex with ML300-Derived noncovalent inhibitors was obtained from the Protein Data Bank (PDB ID: 7LME)^34^. To facilitate the docking process, bound ligands, surrounding water molecules, native zinc ions, B-chain with missing hydrogen atoms, and charge states were removed using PyMOL. Only the A-chain remained, containing 304 amino acid residues (Fig. S1a–d), which was deemed adequate for docking. The A-chain has the full active site of the target protein with a catalytic binding pocket and the subsite binding cleft, including S1, S2, S1′, S4, and S5, as shown in Fig. 3b^35,36^. The ligands used in this in silico study were antcin (A, B, C, H, I, and M), citronellol, limonene, and the positive control compound GC376. The structures of these natural compounds were obtained from the National Center for Biotechnology Information PubChem [https://pubchem.ncbi.nlm.nih.gov]. However, antcin-I and antcin-M are absent from this database. Thus, structures were drawn using CHEM-SPACE https://chem-space.com, as shown in Fig. 1a–c. Lipinski’s rule of five parameters was used to gauge the drug-likeness of all ligands (molecular weight < 500 Da, no more than five hydrogen bond donors, the number of hydrogen bond acceptors should be less than 10, and ClogP should not be greater than 5). Lipinski’s rule of five parameters was evaluated using the admetSAR server2 webtool [http://lmmd.ecust.edu.cn/admetsar2]^33^ as detailed in Table S1. Physicochemical properties and toxicity risk assessment predictions were made via the OSIRIS property explorer software. This was done by uploading the respective compounds’ SMILES into the web server and software.

### Molecular docking between ligands and the target protein

The molecular docking was executed using the improved version of the CB-Dock server for protein–ligand blind docking; [CB-Dock2 https://cadd.labshare.cn/cb-dock2/php/index.php]^27^ which is a highly automatic protein–ligand blind docking process involving 4 steps that include (i) data input, (ii) data processing, (iii) cavity detection and docking, and (iv) visualization and analysis as shown in Fig. S2. (i) The PDB file of SARS-CoV-2-3CL^pro^ and the SDF file of the antcins (A, B, C, H, I, and M), non-antcins (citronellol and limonene), and the positive control compound GC376 were uploaded for data input. (ii) The data processing started with a fixed chain, the removal of water and heterogeneous groups, and the addition of hydrogen for protein preparation using the PyMOL Molecular Graphic System. For ligands, the initial conformation was checked, the 3D conformation was generated to fit the target protein, and hydrogen and charge were added to the ligand, which is greater than FP2. (iii) These searches for protein and ligand templates with less than RSMD 4 Å and FP2 greater than 0.4 Å for the ligands, respectively, provided the output of 5 templates with the best FP2 similarities and produced the optimal binding pose of each site. (iV) This allowed searching for the best cavities of the targeted protein for a selected ligand that may contain an active site but may not be based on the binding affinity energy. It can be selected based on prior knowledge of the catalytic binding site. Therefore, we have chosen ligand and protein complexes in which the ligand was bound at the active site of the target protein with the catalytic binding pocket and the subsite binding cleft, including S1, S2, S1′, S4, and S5, as shown in Fig. 3b^35,36^ and checked their binding affinity energy (kcal/mol) of the tested compounds with 3CL^Pro^. CB-Dock2 used docking score as a binding affinity energy (kcal/mol). Then, we eliminated ligands bound other than the subsite binding cleft. The 3-dimensional coordinates of the protein and ligands' volume (Å3) and grid box were generated automatically [center size (x, y, z), and docking size (x, y, z)], and ligand-residue contacts were also summarized (see Table 1). Furthermore, the protein complex, which had the lowest binding affinity energy and contact with the active site of the protein, was selected for redocking using the Auto-Dock Vina module; UCSF-Chimera (version 1.16) and used the grid box generated by CB-Dock2 to confirm and provide similar results as shown in Table 1. The interaction of protein and ligand and the detailed protein–ligand complex were visualized and analyzed using PyMOL.

### Validation of the molecular docking results and interaction

To ensure accurate results, the docking protocol was validated by using it to perform a re-docking calculation. Here, the goal is to correctly reproduce the binding pose and the molecular interactions of the co-crystallized ligand in the active site of the experimentally crystallized protein structure. Thus, all native ligands and target proteins were separated and prepared for docking via UCSF-Chimera (version 1.16). After docking, the protein–ligand interactions were evaluated and visualized using the Protein*Plus* web server by uploading the complex PDB format [https://proteins.plus] that can calculate the binding site within a minute binding site using binding detection site based on drug score and pocked volume [Å^3^] similarly detected binding pocket and the subsite binding cleft, including S1, S2, S1′, S4, and S5, as shown in Fig. 3b^35,36^. Ligand–protein complex was uploaded and used (PoseView), which provides the detailed interaction between the ligand and protein complex in 2D form but only shows hydrogen and hydrophobic interaction. (Protoss) was performed to check their binding site [http://proteins.plus]^30^. The detailed interaction is provided in Fig. S3a–h. Furthermore, the Protein–Ligand Interaction Profiler (PLIP) [https://plip-tool.biotec.tu-dresden.de/plip-web/plip/index] for identifying the non-covalent interactions between biological macromolecules and their ligands^29^ which also offers pi-cation interactions, salt bridges, water bridges, and halogen bonds. These results were visualized using PyMOL. To facilitate the interaction, hydrogen was added to the ligand using PyMOL. We run the (PLIP) for detailed interactions, including pi-cation interactions, salt bridges, hydrogen bonds, and hydrophobic and halogen bonds.

### Molecular dynamics (MD) simulation

We performed molecular dynamics (MD) simulations using Maestro-Desmond implemented by Schrödinger to gain further insights into the molecular docking results. After the docking calculations, the resulting ligand–protein complexes were used as initial structures for the MD simulations. For preprocessing, the protein preparation wizard in Maestro was used to add missing hydrogens and side chains. Additionally, the bond order of the ligands was corrected, and the complexes were charged with the OPLS3e force field using Maestro's system builder^57^. To ensure an accurate representation of the system, the ligand–protein complexes were immersed in an SPC (simple point charge)^58^ water box, with a buffer extending 10 Å beyond the complex's atoms to prevent the protein from leaving the water box. To mimic physiological conditions, 0.15 M NaCl was added to the solution. The amount of Na^+^ and Cl^−^ ions that needed to be added for this was determined automatically. To balance the charges of the systems, 3 additional Na^+^ ions were added in the cases of Citronellol and Limonene, while in all other cases, 4 Na^+^ ions were added to neutralize the system. The MD simulations were performed under constant temperature (300 K) and pressure (1 atm) conditions, utilizing the NPT ensemble to maintain a constant number of particles throughout the simulation. The OPLS3e force field was employed for all simulations. The protein–ligand complex was simulated for 100 ns in each case, with data recorded every 4.8 ps. Plots and figures depicting the simulations were generated using Maestro's Desmond simulation interaction diagram tool.

### Enzymatic activity assay

In vitro, inhibition of the 3CLPro activity was measured using a commercially available enzymatic activity assay kit (SensoLyte 520 SARS-CoV-2 3CL Protease Activity Assay Kit, ANASPEC, Fremont, CA, USA). The experiment was performed according to the manufacturer’s instructions, and all samples were assayed in triplicate. An excitation wavelength of 360 nm was used, and fluorescence was measured at an emission wavelength of 520 nm based on the presence of a fluorogenic substrate and an excitation wavelength of 490 nm. The non-covalent inhibitor GC376 was provided as an internal positive drug control and was used at a concentration of 100 nM in the assay. DMSO was used as the vehicle control and substrate, with enzyme only as the negative control. Natural compounds such as antcin (A, B, C, H, I, and M), citronellol, and limonene at specific concentrations were tested for the potential inhibition of the 3CLPro activity, as shown in Fig. 5. The compounds with 99% purity were obtained from the R&D Center of Taiwan Leader Biotechnology Corp (Taichung, Taiwan). Each compound's stock solutions were stored at − 20 °C until use. Serial doubling dilutions yielded a final concentration of 1.25 to 20 μM for assaying the antcin-B compound. Readings (absolute fluorescence values at 1200 grain) were made using a Fluoro-luminous-Photometer plate Chameleon microplate reader (HIDEX, Turku, Finland). The percentage (%) of inhibition of 3CL^Pro^ activity was calculated as the ratio of activity in the presence of the inhibitor and total activity, considering background.

### Statistical analysis

Data are expressed as mean ± SD. All data were analyzed using GraphPad Prism version 9.5.0 statistical software for Apple M1 (GraphPad Software, San Diego, CA, USA). Statistical analysis was performed using one-way ANOVA followed by Dunnett’s test for multiple comparisons. *P*-values of less than 0.05 *, 0.01 **, 0.001 ***, and 0.0001 **** were considered statistically significant for the sample treatment group vs. the control group.

### Supplementary Information

Supplementary Information.

## Data Availability

All datasets included in the study are available from the corresponding author upon request.
