# Supplementary material for: Antcin-B, a phytosterol-like compound from Taiwanofungus camphoratus inhibits SARS-CoV-2 3-chymotrypsin-like protease (3CLPro) activity in silico and in vitro
Source: Sci Rep. 2023 Oct 10;13:17106. doi: 10.1038/s41598-023-44476-x (PMC10564890; doi:10.1038/s41598-023-44476-x)
Supplement: Supplementary file 1 — Supplementary Information. [file 41598_2023_44476_MOESM1_ESM.docx]

**Antcin-B, a phytosterol-like compound from *Taiwanofungus camphoratus* inhibits SARS-CoV-2 3-chymotrypsin-like protease (3CL^Pro^) activity *in-silico* and *in vitro***

**Gyaltsen Dakpa^1,2^, K. J. Senthil Kumar^3^, Jochem Nelen^4^, Horacio Pérez-Sánchez^4^ & Sheng-Yang Wang^1,5,6,7,*^**

^1^Molecular and Biological Agricultural Sciences Program, Taiwan International Graduate Program, Academia Sinica, Taipei-108, Taiwan. ^2^Graduate Institute of Biotechnology, National Chung Hsing University, Taichung-402, Taiwan. ^3^Bachelor Program of Biotechnology, National Chung Hsing University, Taichung-402, Taiwan. ^4^Structural Bioinformatics and High-Performance Computing Research Group (BIO-HPC), HiTech Innovation Hub, Universidad Católica de Murcia (UCAM), 30107 Murcia, Spain. ^5^Department of Forestry, National Chung Hsing University, Taichung-402, Taiwan. ^6^Special Crop and Metabolome Discipline Cluster, Academy of Circle Economy, National Chung Hsing University, Taichung-402, Taiwan. ^7^Agricultural Biotechnology Research Center, Academia Sinica, Taipei-108, Taiwan. Email: taiwanfir@drgon.nchu.edu.tw


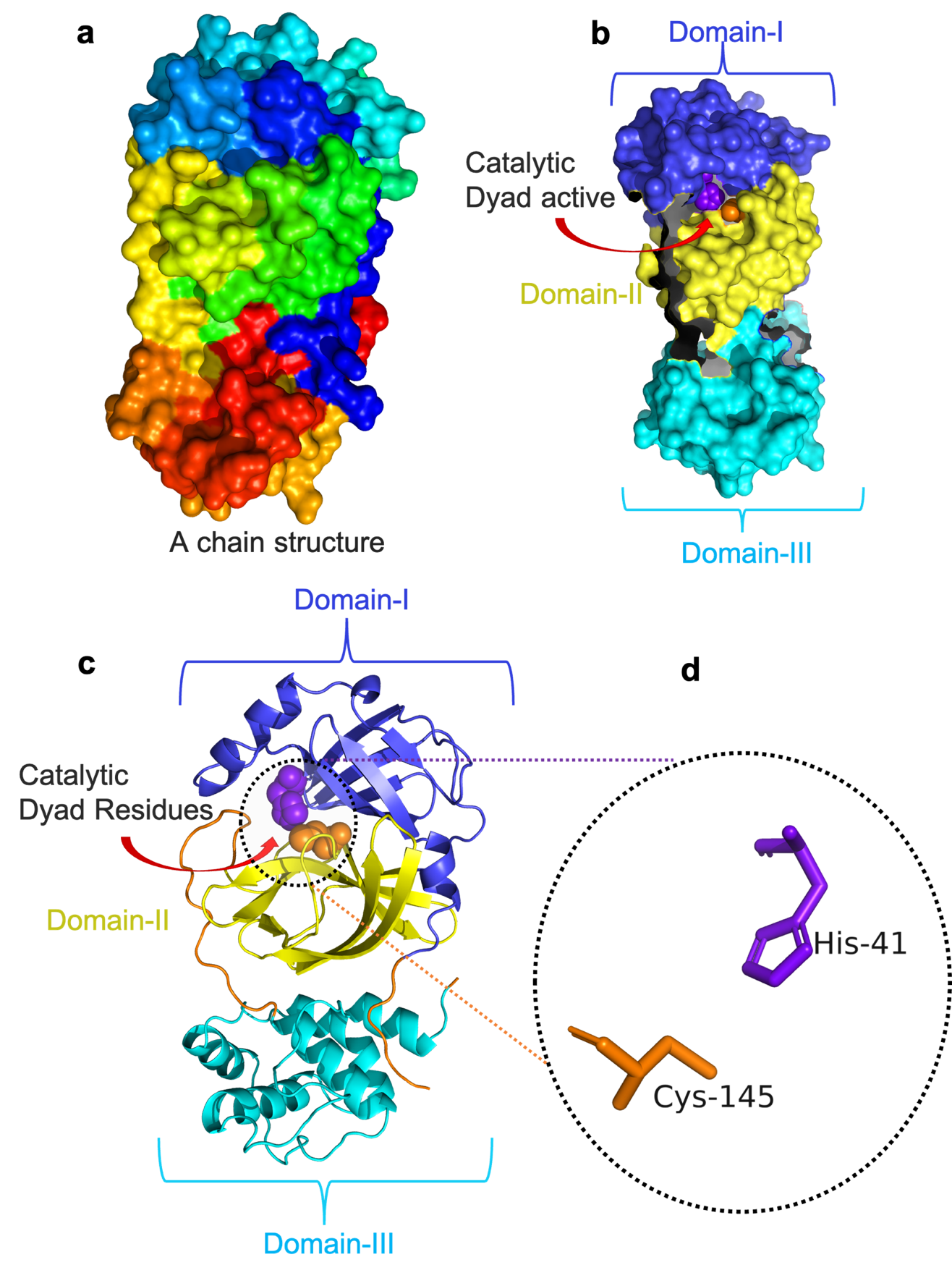


**Figure S1.** Three-dimensional structure of SARS-CoV-2 3CL^pro^. **(a)** 3D structure of SAR-Cov-2-3CL^pro^ represented in a surface model with a rainbow color which was obtained from the RCSB Protein Data Bank (PDB) with (PDB ID; 7LME)^1^ and visualized and only-A was retained using the PyMoL Molecular Graphic System GL_VERSION: 2.5.4, which contains the 304 amino acid residues. **(b)** 3D surface model containing three domains of 3CL^Pro^ domain-I indicated in deep blue color; domain-ii, shown in yellow color; and domain-III, represented in cyan color. The central catalytic dyed residues (His41 and Cys145) are presented in a sphere model with purple and orange colors, respectively. **(c)** three domains of 3CL^Pro^ are shown in the cartoon model with three colors which are the same as in B. **(d)** then catalytic dyed residues are present in the sticks model with specific positive residues in the 3CL^pro^ chain, that is, His41 and Cys145.


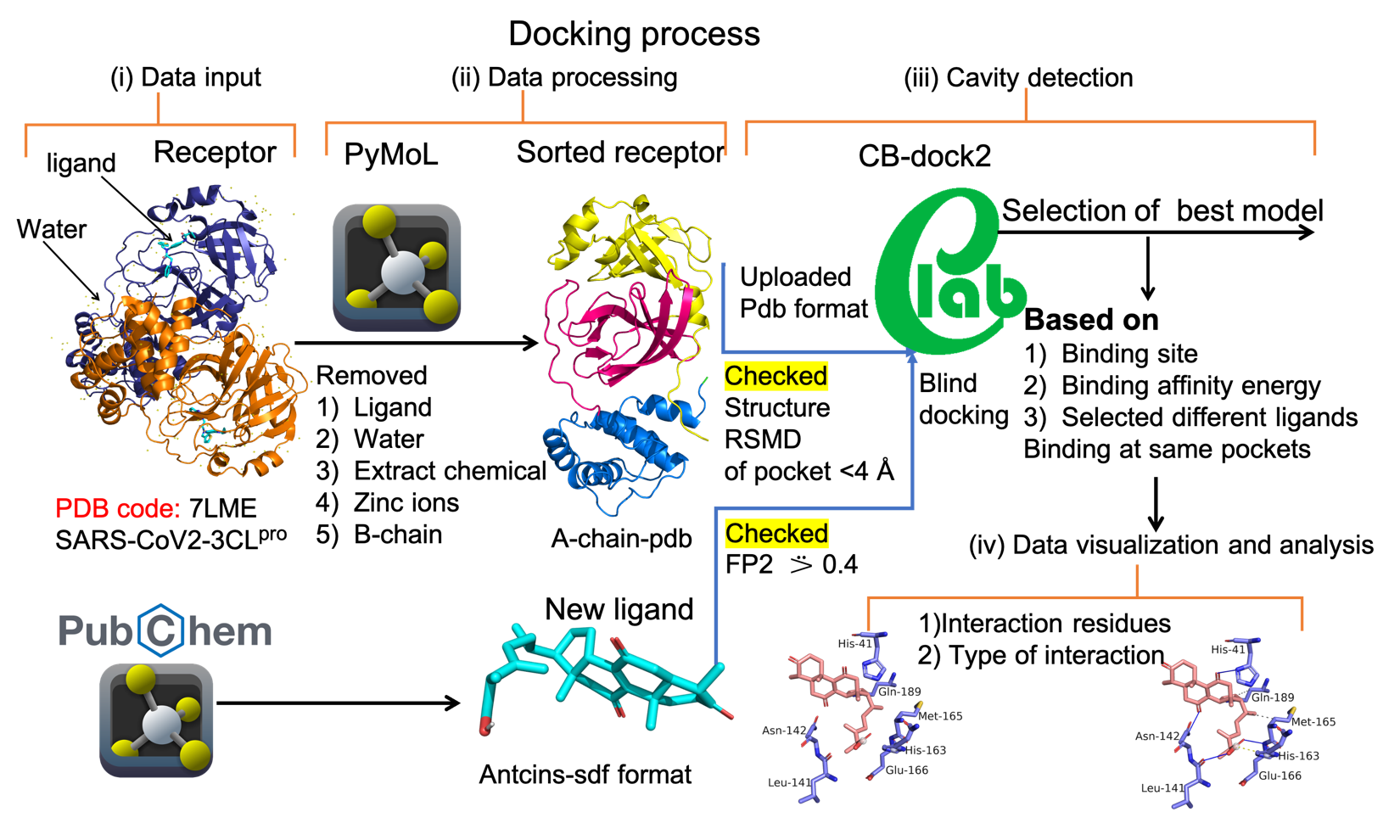


**Figure S2. Molecular docking process using the CB-Dock2 as a technique**

The molecular docking was executed using the improved version of the CB-Dock server for protein-ligand blind docking; CB-Dock2 https://cadd.labshare.cn/cb-dock2/php/index.php^2^ which is highly automatic protein-ligand blind docking by 4 steps that include (i) data input, (ii) data processing, (iii) cavity detection and docking, and (iv) visualization and analysis. This docking predicted more than 85% accuracy compared to their docking techniques, including FitDock, MTiAutoDock, SwissDock, and COACH-Dn^2^. The details are shown in the figure. The data process was employed with the help of the PyMOL Molecular Graphic System to remove the water, b-chain, Zinc Ions, and water to facilitate the docking process.


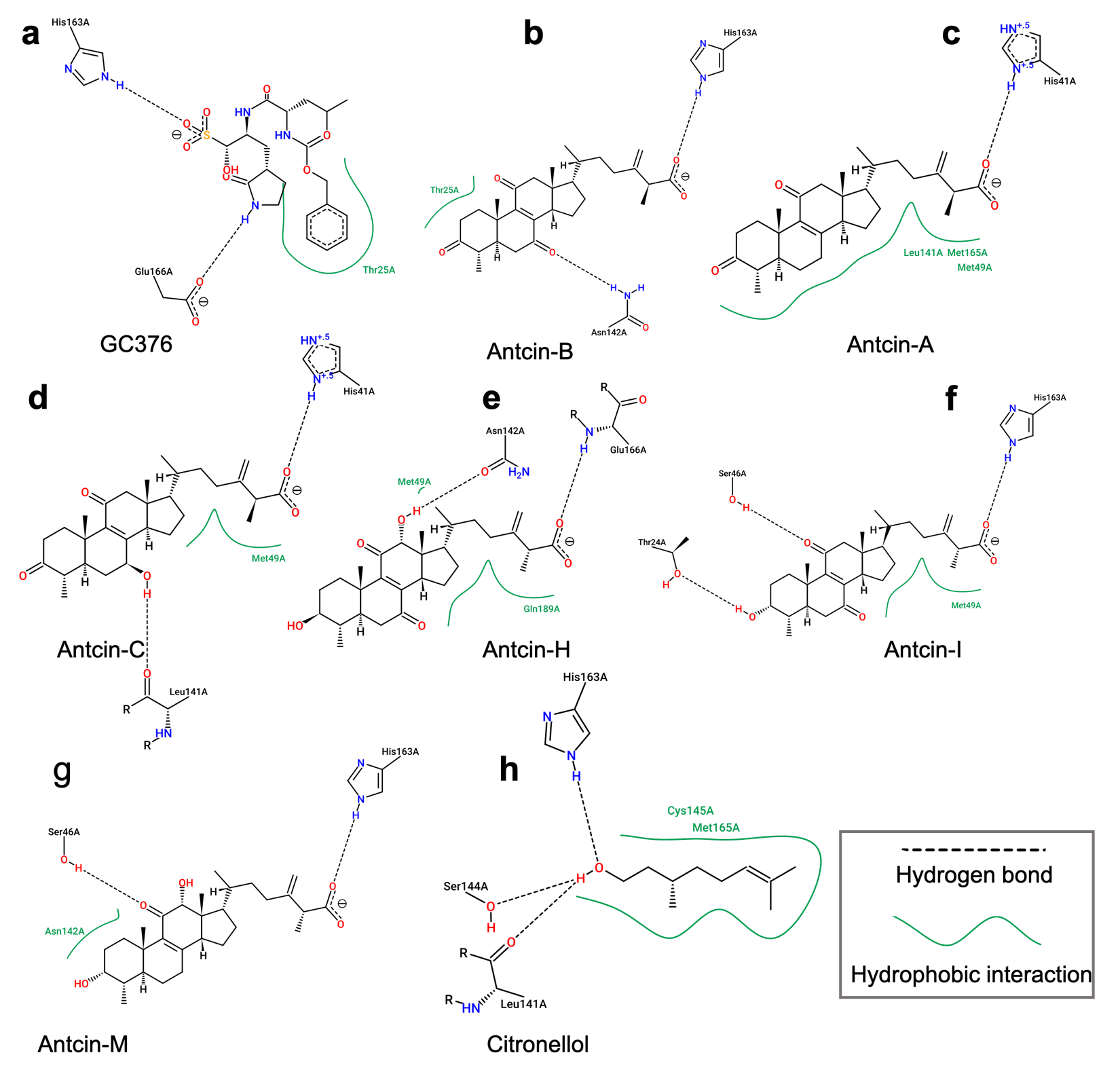


**Figure. S3** 2D protein-ligand interaction diagrams obtained by ProteinPlus. Further validating docking results and interactions, we have performed the ProteinPlus, which provides the details of chemical 2D protein–ligand interaction diagrams. These pre-processing tasks like structure quality assessment (EDIA), hydrogen placement (Protoss), and the search for alternative conformations (SIENA). Beyond that, it also addresses frequent problems such as generating 2D-interaction diagrams (PoseView), http://proteins.plus. The complex interaction between antcins, non-antcins, and GC376 and SARS-CoV2-3CL^Pro^ inhibits the activity that can be a potential leading drug candidate to protect from COVID-19. (a) GC376, (b) antcin-A, (c) antcin-B, (d) antcin- C, (e) antcin-H, (f) antcin-I, (g) antcin-M, (h) citronellol, are interaction with residues of SARS-CoV2-3CL^Pro^ are represented in the 2D visualization. Interaction between residues and drugs depicts all structure diagrams following the IUPAC drawing conventions. The black dishes line indicated the hydrogen bonds and the green lines represented hydrophobic interaction. The formation of hydrogen bonds is detailed depicted in. It was observed that the drugs interacted with neighboring amino acid residues. Based on the ProteinPlus, there is no interaction of Limonene with 3CL^Pro^ due to a lack of hydron bonds and to further of limonene from 3CL^Pro^ to be hydrophobic.


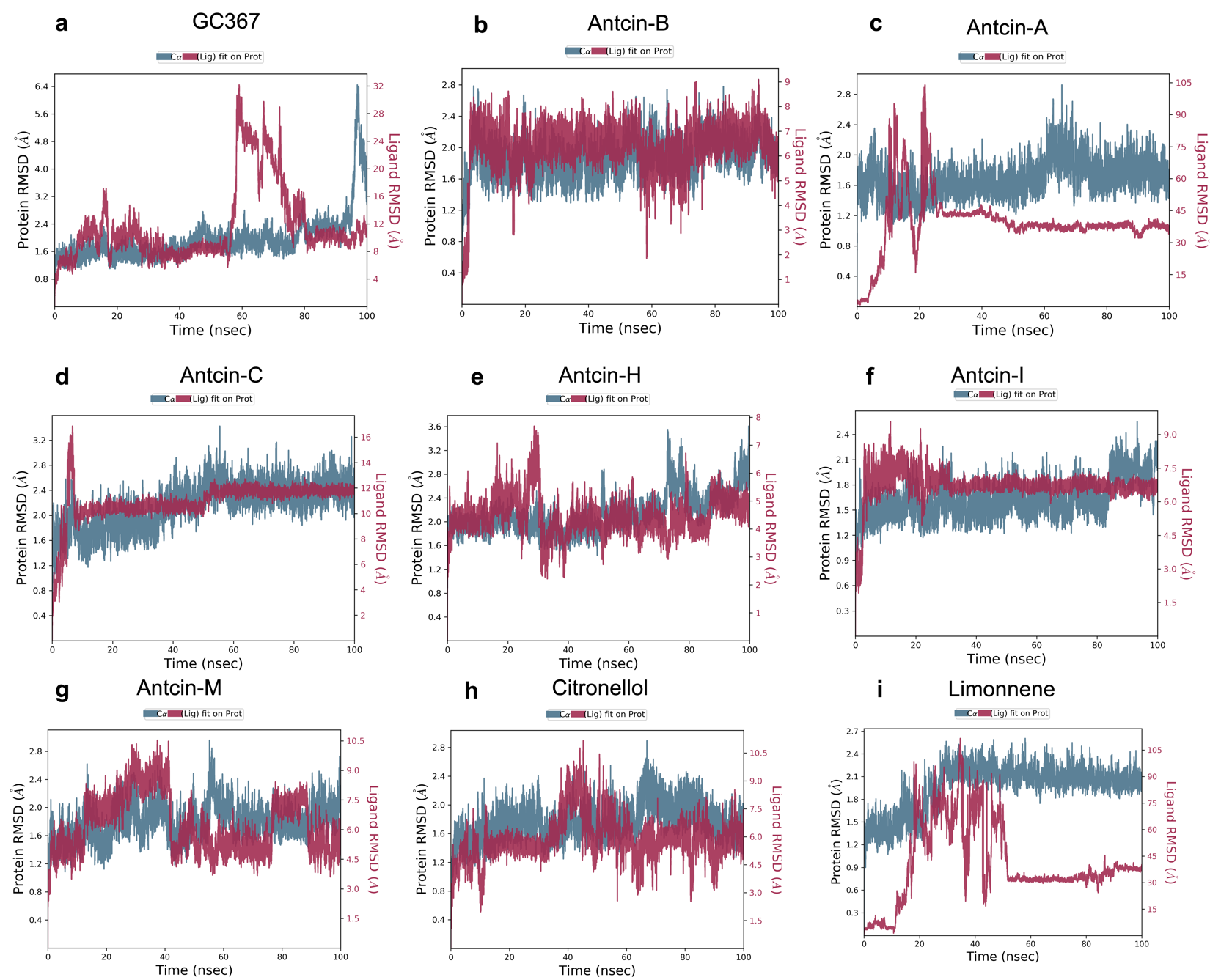


**Figure S4**. Root-mean-square-deviation (RMSD)  of the protein-ligand complex during the 100ns MD simulation for the complexes of 3CL^pro^ with: (a) GC376, (b) Antcin-B, (c) Antcin-A, (d) Antcin-C, (e) Antcin-H, (f) Antcin-I, (g) Antcin-M, (h) Citronellol and (i) Limonene. The blue graphs correspond to the protein RMSD, and the red graphs correspond to the ligand RMSD. Usually, low ligand RMSD can indicate the formation of a stable complex.


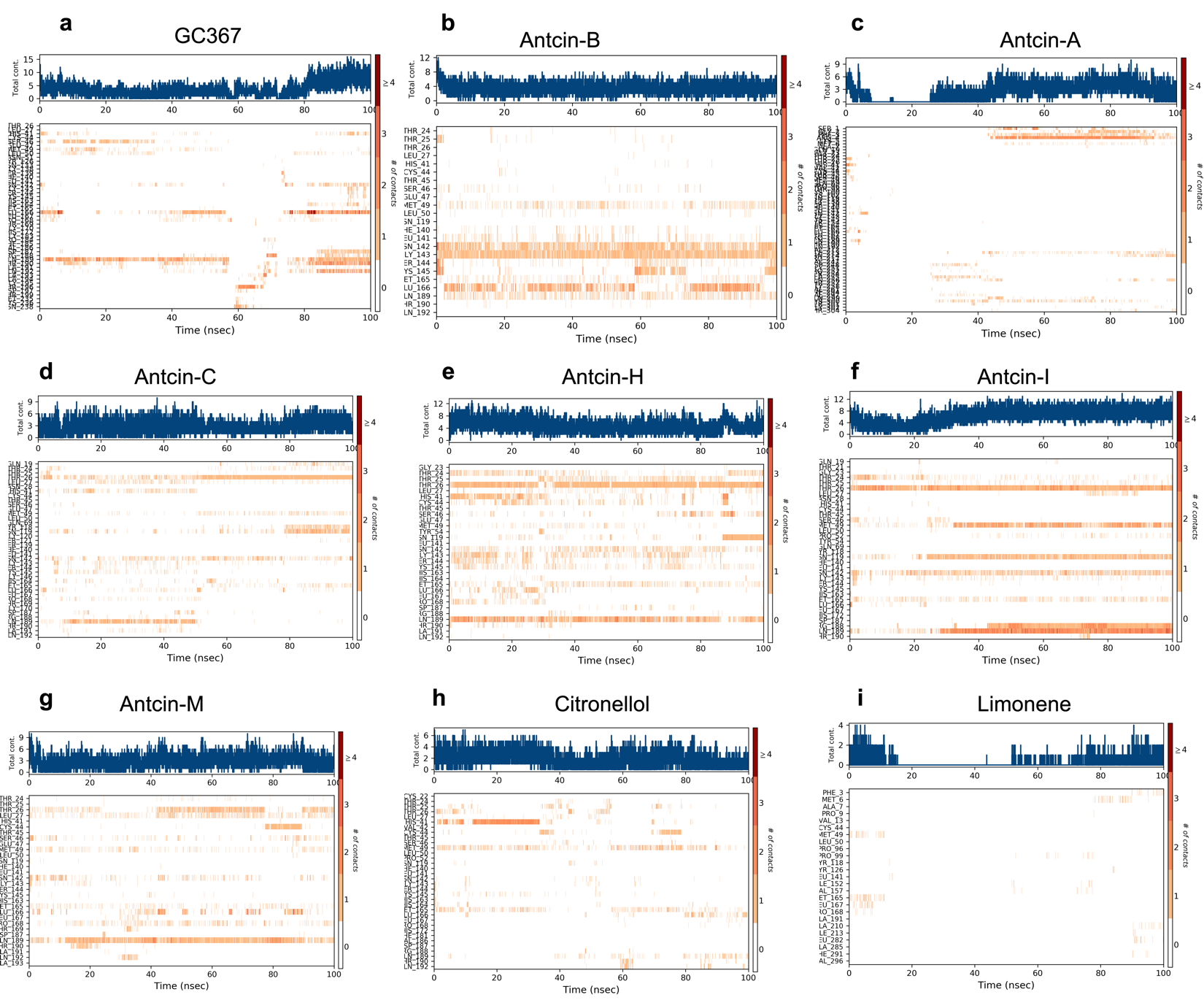


**Figure S5. Protein–ligand contact timeline plot during 100 ns MD simulation.** Total contact timeline plot of the tested ligands bound to the active inhibitory site at 3CLPpro protein during the 100 ns MD simulation, which is (a) GC376, (b) antcin-A, (c) antcin-B, (d) antcin-C, (e) antcin- H, (f) antcin-I, (g) antcin-M, (h) citronellol, and (i) limonene.


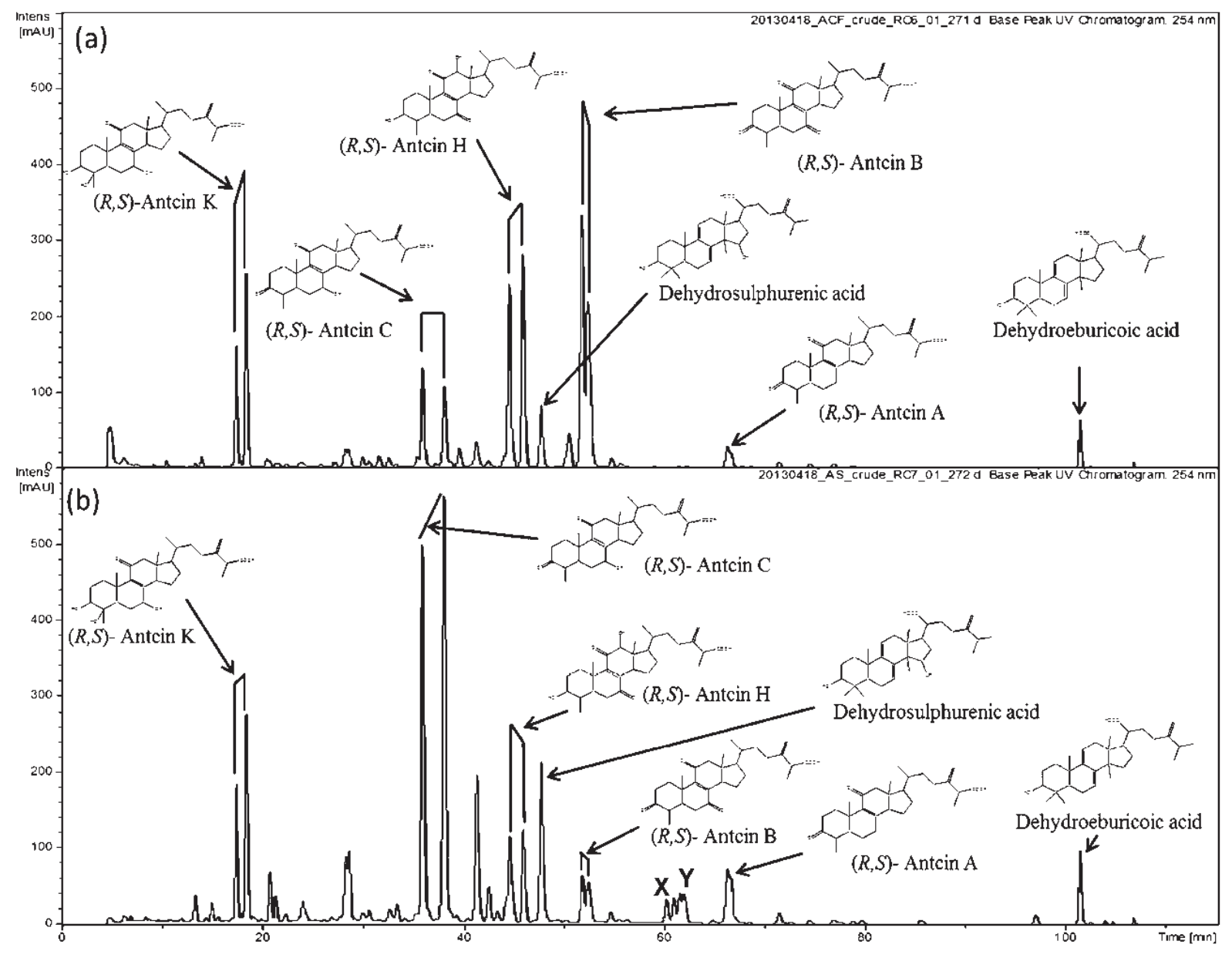


**Figure S6.** HPLC profiling of metabolites of Taiwanofungus camphoratus (a) and A. salmonea (b) fruiting bodies^3^

**Table S1.** ADMET properties of best hits compounds with GC376, ADMET was predicted using the AdmetSAR server2

| **Class** | **Properties** | **Antcin-A** | **Antcin-B** | **Antcin-C** | **Antcin-H** | **Antcin-I** | **Antcin-M** | **Citrollelol** | **Limonene** | **GC376** |
| --- | --- | --- | --- | --- | --- | --- | --- | --- | --- | --- |
| **Absoprtion** | Caco-2 Permeability | −5.231 | −4.977 | −4.997 | −5.235 | −5.031 | −5.055 | −4.252 | −4.32 | −16.11 |
|  | MDCK Permeability | 1.3x10^5^ | 1.6X10^5^ | 1.3x10^5^ | 6.7X10^5^ | 2.8x-0^5^ | 3.7-10^5^ | 2x0^5^ | 1.9x10^5^ | 0.0001 |
|  | Pgp-inhibitor | 0.259 | 0.01 | 0.459 | 0.485 | 0.004 | 0.959 | 0.006 | 0.002 | 0.001 |
|  | Pgp-substrate | 0.004 | 0.007 | 0.003 | 0.12 | 0.379 | 0.005 | 0.006 | 0 | 0.102 |
|  | HIA | 0.039 | 0.138 | 0.152 | 0.044 | 0.212 | 0.017 | 0.005 | 0.003 | 0.99 |
| **Distribution** | PPB | 95.86% | 96.40% | 95.88% | 95.23 | 96.92% | 96.30% | 93.47% | 86.38% | 58.82% |
|  | BBB | 0.835 | 0.799 | 0.848 | 0.41 | -0.806 | 0.201 | 0.948 | 0.989 | 0.341 |
| **Metabolism** | CYP1A2 inhibition | 0.01 | 0.006 | 0.007 | 0.013 | 0.009 | 0.046 | 0.65 | 0.678 | 0.006 |
|  | CYP1A2 substrate | 0.619 | 0.602 | 0.591 | 0.593 | 0.495 | 0.624 | 0.427 | 0.652 | 0.039 |
|  | CYP2C19 inhibitor | 0.016 | 0.01 | 0.01 | 0.012 | 0.008 | 0.021 | 0.092 | 0.223 | 0.035 |
|  | CYP2C19 substrate | 0.904 | 0.838 | 0.883 | 0.882 | 0.882 | 0.878 | 0.604 | 0.834 | 0.057 |
|  | CYP2C9 inhibitor | 0.169 | 0.06 | 0.078 | 0.044 | 0.046 | 0.113 | 0.052 | 0.06 | 0.056 |
|  | CYP2C9 substrate | 0.7 | 0.271 | 0.472 | 0.673 | 0.497 | 0.708 | 0.765 | 0.804 | 0.915 |
|  | CYP2D6 inhibitor | 0.002 | 0.003 | 0.001 | 0.002 | 0.002 | 0.002 | 0.007 | 0.02 | 0.003 |
|  | CYP2D6 substrate | 0.727 | 0.261 | 0.297 | 0.139 | 0.185 | 0.178 | 0.115 | 0.874 | 0.127 |
|  | CYP3A4 inhibitor | 0.206 | 0.151 | 0.072 | 0.076 | 0.135 | 0.143 | 0.018 | 0.057 | 0.025 |
|  | CYP3A4 substrate | 0.23 | 0.222 | 0.174 | 0.327 | 0.247 | 0.33 | 0.162 | 0.253 | 0.055 |
| **Toxicity** | Human Hepatotoxicity | 0.316 | 0.461 | 0.453 | 0.745 | 0.612 | 0.568 | 0.573 | 0.69 | 0.07 |
|  | Drug Induced Liver Injury | 0.548 | 0.552 | 0.523 | 0.157 | 0.122 | 0.219 | 0.028 | 0.037 | 0.03 |
|  | carcinogens | 0.067 | 0.095 | 0.053 | 0.092 | 0.0078 | 0.194 | 0.224 | 0.922 | 0.023 |
|  | corrosives | 0.003 | 0.003 | 0.003 | 0.003 | 0.004 | 0.005 | 0.915 | 0.849 | 0.003 |
|  | irritants | 0.009 | 0.01 | 0.009 | 0.012 | 0.01 | 0.018 | 0.985 | 0.981 | 0.007 |
|  | Sensitizer | 0.017 | 0.01 | 0.012 | 0.061 | 0.026 | 0.442 | 0.857 | 0.355 | 0.125 |
|  | Acute oral toxicity  (kg/ mol) | 0.889 | 0.931 | 0.968 | 0.774 | 0.795 | 0.497 | 0.01 | 0.017 | 0.03 |

NOTE: The Madin-Darby canine kidney (MDCK) permeability, low permeability: < 2 × 10^−6^ cm/s, medium permeability: 2–20 × 10^−6^ cm/s, and high passive permeability: > 20 × 10^−6^ cm/s. Caco-2 Permeability: higher than -5.15 Log unit. Human intestinal absorption (HIA), HIA+( HIA < 30%); Category 0: HIA-( HIA < 30%) and Blood-brain barrier (BBB), If PPB is higher than 90% moderate, 100 % is high, and over 100% is very high. Acute oral toxicity (kg/ mol) Category 0: low toxicity; Category 1: high toxicity.

**Table S2.** Druglikeness prediction according to Lipinski’s rule of antcins (A, B, C, H, I, and M), non-antcins (citronellol and limonene ), and GC376 using the admetSAR server2

| **Properties** | **Antcin-A** | **Antcin-B** | **Antcin-C** | **Antcin-H** | **Antcin-I** | **Antcin-M** | **Citronellol** | **Limonene** | **GC376** |
| --- | --- | --- | --- | --- | --- | --- | --- | --- | --- |
| **Molecular Weight** | 454 | 468.3 | 470.3 | 486.3 | 470.3 | 472.32 | 156.15 | 136.1 | 485.2 |
| **Solubility  (Log S)** | −5.6 | −5.26 | −5.21 | −4.81 | −5.11 | −5.34 | −2.15 | −2.54 | −2.75 |
| **TPSA (Å)** | 71.4 | 88.51 | 91.67 | 111.9 | 91.67 | 91.67 | 20.23 | 0 | 182.3 |
| **cLOgP** | 4.58 | 5.49 | 5.35 | 4.5 | 4.94 | 5.22 | 3.35 | 3.36 | -2.21 |
| **H-Bond  Acceptors** | 4 | 5 | 5 | 6 | 5 | 5 | 1 | 0 | 11 |
| **H-Bond  Donor** | 1 | 1 | 1 | 2 | 2 | 2 | 1 | 0 | 5 |
| **Druglikeness** | −1.85 | −1.85 | −1.44 | −1.03 | −1.78 | −2.42 | −8.68 | −21.85 | −33.9 |
| **Drug score** | 0.19 | 0.22 | 0.24 | 0.31 | 0.41 | 0.22 | 0.16 | 0.35 | 0.37 |
| **Mutagenic** | no | no | no | no | no | no | no | no | no |
| **Tumorigenic** | no | no | no | no | no | no | no | no | no |
| **Reproductive  effect** | no | no | no | no | no | no | no | no | no |
| **Irritants** | no | no | no | no | no | no | no | no | no |
| **Lipinski  Rule** | Accepted | Accepted | Accepted | Accepted | Accepted | Accepted | Accepted | Accepted | Accepted |
| **Pfizer Rule** | Rejected | Accepted | Accepted | Accepted | Accepted | Accepted | Rejected | Rejected | Accepted |
| **GSK Rule** | Rejected | Rejected | Rejected | Rejected | Rejected | Rejected | Accepted | Rejected | Rejected |
| **Golden triangle rule** | Accepted | Accepted | Accepted | Accepted | Accepted | Accepted | Rejected | Rejected | Accepted |

**Lipinski Rule**: MW ≤ 500; logP ≤ 5; Hacc ≤ 10; Hdon ≤ 5 are acceptable.

**Pfizer Rule**:logP > 3; TPSA < 75, Compounds with a high log P (>3) and low TPSA (<75) are likely to be toxic.

**Golden triangle rule** 200 ≤ MW ≤ 50; -2 ≤ logD ≤ 5, are acceptable.

**References**

1 Han, S. H. *et al.* Structure-based optimization of ML300-derived, noncovalent inhibitors targeting the severe acute respiratory syndrome coronavirus 3CL protease (SARS-CoV-2 3CL(pro)). *J Med Chem* **65**, 2880-2904 (2022). https://doi.org:10.1021/acs.jmedchem.1c00598

2 Liu, Y. *et al.* CB-Dock2: improved protein-ligand blind docking by integrating cavity detection, docking and homologous template fitting. *Nucleic Acids Res* **50**, W159-164 (2022). https://doi.org:10.1093/nar/gkac394

3 Chen, C. Y. *et al.* Metabolite profiling and comparison of bioactivity in *Antrodia cinnamomea* and *Antrodia salmonea* fruiting bodies. *Planta Med* **82**, 244-249 (2016). https://doi.org:10.1055/s-0035-1558141
